# In vivo non-invasive near-infrared spectroscopy distinguishes normal, post-stroke, and botulinum toxin treated human muscles

**DOI:** 10.1038/s41598-021-96547-6

**Published:** 2021-09-03

**Authors:** Antonio Currà, Riccardo Gasbarrone, Alessandra Cardillo, Francesco Fattapposta, Paolo Missori, Lucio Marinelli, Giuseppe Bonifazi, Silvia Serranti, Carlo Trompetto

**Affiliations:** 1 Academic Neurology Unit, A. Fiorini Hospital, Via Firenze snc, 04019 Terracina, LT Italy; 2 Research Center for Biophotonics, Sapienza University of Rome, Latina, Italy; 3 Department of Chemical Engineering, Materials and Environment, Sapienza University of Rome, Rome, Italy; 4 Neurology Unit, Policlinico Umberto I, Department of Human Neurosciences, Sapienza University of Rome, Rome, Italy; 5 Neurosurgery Unit, Policlinico Umberto I, Department of Human Neurosciences, Sapienza University of Rome, Rome, Italy; 6 Department of Neuroscience, IRCCS Ospedale Policlinico San Martino, Genoa, Italy; 7 Department of Neuroscience, Rehabilitation, Ophthalmology, Genetics, Maternal and Child Health, University of Genoa, Genoa, Italy

**Keywords:** Stroke, Predictive markers, Near-infrared spectroscopy

## Abstract

In post-stroke hemiparesis, neural impairment alters muscle control, causing abnormal movement and posture in the affected limbs. A decrease in voluntary use of the paretic arm and flexed posture during rest also induce secondary tissue transformation in the upper limb muscles. To obtain a specific, accurate, and reproducible marker of the current biological status of muscles, we collected visible (VIS) and short-wave Infrared (SWIR) reflectance spectra in vivo using a portable spectroradiometer (350–2500 nm), which provided the spectral fingerprints of the elbow flexors and extensors. We compared the spectra for the affected and unaffected sides in 23 patients with post-stroke hemiparesis (25–87 years, 8 women) and eight healthy controls (33–87 years, 5 women). In eight patients, spectra were collected before and after botulinum toxin injection. Spectra underwent off-line preprocessing, principal component analysis, and partial least-squares discriminant analysis. Spectral fingerprints discriminated the muscle (biceps vs. triceps), neurological condition (normal vs. affected vs. unaffected), and effect of botulinum toxin treatment (before vs. 30 to 40 days vs. 110 to 120 days after injection). VIS-SWIR spectroscopy proved valuable for non-invasive assessment of optical properties in muscles, enabled more comprehensive evaluation of hemiparetic muscles, and provided optimal monitoring of the effectiveness of medication.

## Introduction

Muscles are composed of muscular, connective, vascular, fat, and nervous tissues, and contract to maintain posture or to produce movement^1^. In patients affected by post-stroke hemiparesis, both posture and movement are altered by upper motor neuron syndrome (UMNS) as a result of both negative (weakness and loss of dexterity)^2^ and positive phenomena (increased jerks, spasticity, clonus, spastic dystonia, muscle spasms, and co-contraction)^3^. In the upper limb, a decrease in voluntary use of the paretic arm and flexed posture during rest^4^ promote secondary changes in muscles, mainly represented by increased connective tissue and reduced sarcomere number^5^. Therefore, the affected muscles of stroke patients are impaired by neural signs and structural changes, both of which alter the motor function and lead to disability.

Muscles are examined clinically by palpation and evaluation of passive movements to explore the muscle structure and by assessing passive and active movements (either freely or against resistance) to explore neural impairment (i.e., UMNS signs). To measure strength^6^ and tone^7^, clinical scales are available that basically match the strength and proprioception of the examiner with the active or passive resistance offered by the patient’s limb to displacement^8^. Objective assessment of the muscle structure involves both non-invasive (e.g., ultrasound^9,10^ or magnetic resonance-based imaging techniques^11^) and invasive procedures (e.g., biopsy along with macroscopic and microscopic analysis)^12^. Objective assessment of UMNS signs is a matter of clinical neurophysiology^13^ by means of EMG, ENG, and reflex studies, and biomechanics by analysis of kinematics, limb impedance, and torque to mechanical perturbation or voluntary movement^14^.

Unfortunately, such a comprehensive investigation of muscles is not feasible in clinical practice because it can require specific expertise, devices, and time, as well as cooperation that is hard to elicit in patients, especially children. Having a unique and reliable indicator of the overall state of the muscle that complements both neural and non-neural contributing factors, and that provides reproducible measures that can be replicated over time, would provide equally important information to proceed rationally in planning a patient’s treatment.

Recently, we set up a procedure to determine a marker of the current biological state of the living muscle^15^. It is a non-invasive, photonic method that can be applied bedside without significant cost or time penalties. A small, portable spectrophotometer is used to collect reflectance spectra from muscles in the visible (VIS) and short-wave infrared (SWIR) region; these spectra can be studied and classified by the application of specific statistical and pattern recognition packages (i.e., chemometric analysis)^16^, without requiring chemical information from the analysis of molecular bonds. The collected spectra are surrogates for the complex attributes of the organic structures from which they originate, representing the “fingerprint” of the examined sample. This procedure represents an alternative VIS-SWIR spectroscopy application than that aimed to measure real-time non-invasive detection of tissue hemoglobin oxygenation^17^, or to detect the hemoglobin and myoglobin content of skeletal muscle^18^. In our previous study in healthy subjects^15^, VIS-SWIR reflectance spectra were able to distinguish the upper limb flexors from extensors and were sensitive to anthropometric variables, such as gender, age, and body mass index.

In the present study, we applied VIS-SWIR reflectance spectroscopy in patients with post-stroke hemiparesis. We tested whether the optical properties can discriminate the muscles in healthy subjects from those in patients on the affected and unaffected sides. Moreover, we evaluated whether the optical properties of affected muscles change before and after treatment with botulinum toxin.

## Results

**Figure 1:**
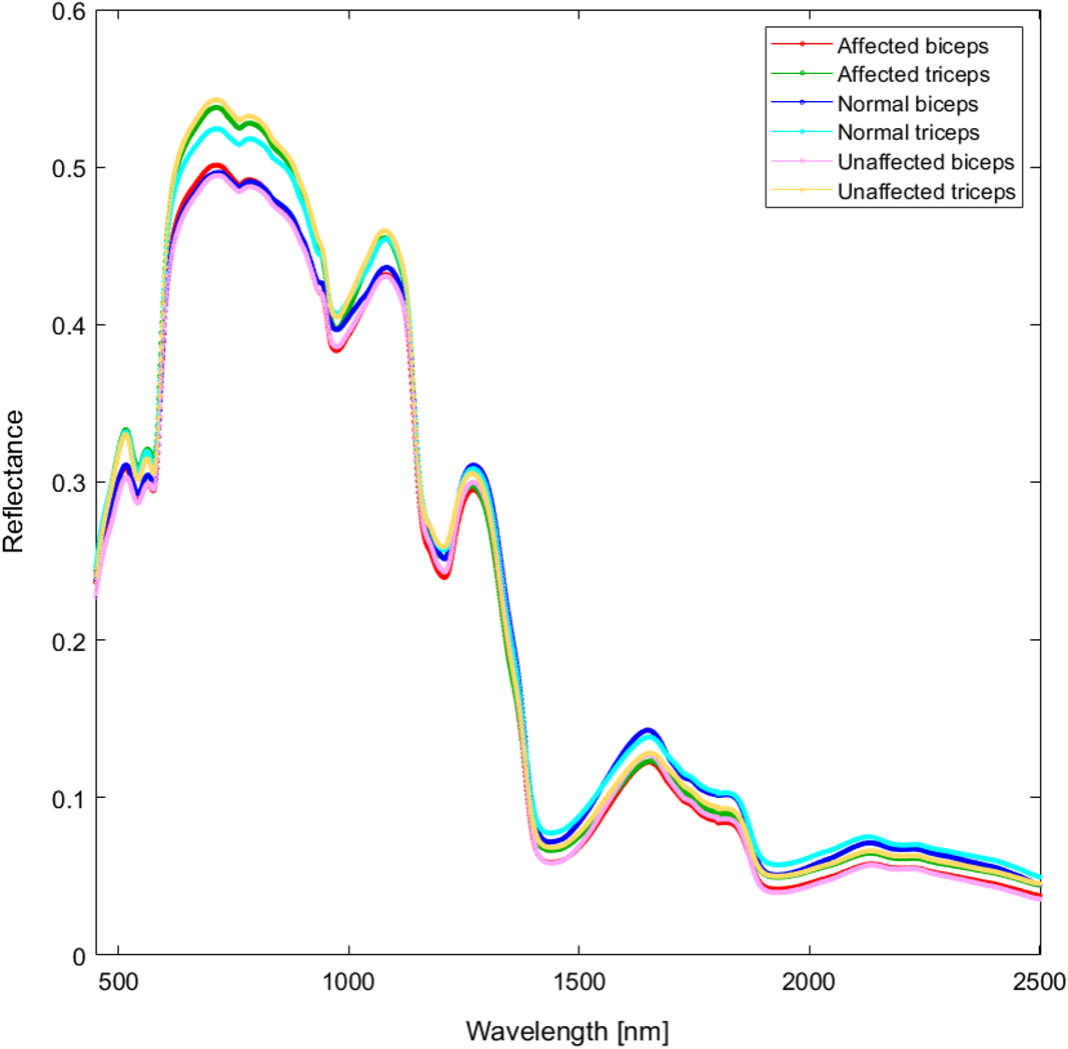
Average reflectance spectra of normal biceps, affected biceps, unaffected biceps, normal triceps, affected triceps, and unaffected triceps.

**Figure 2:**
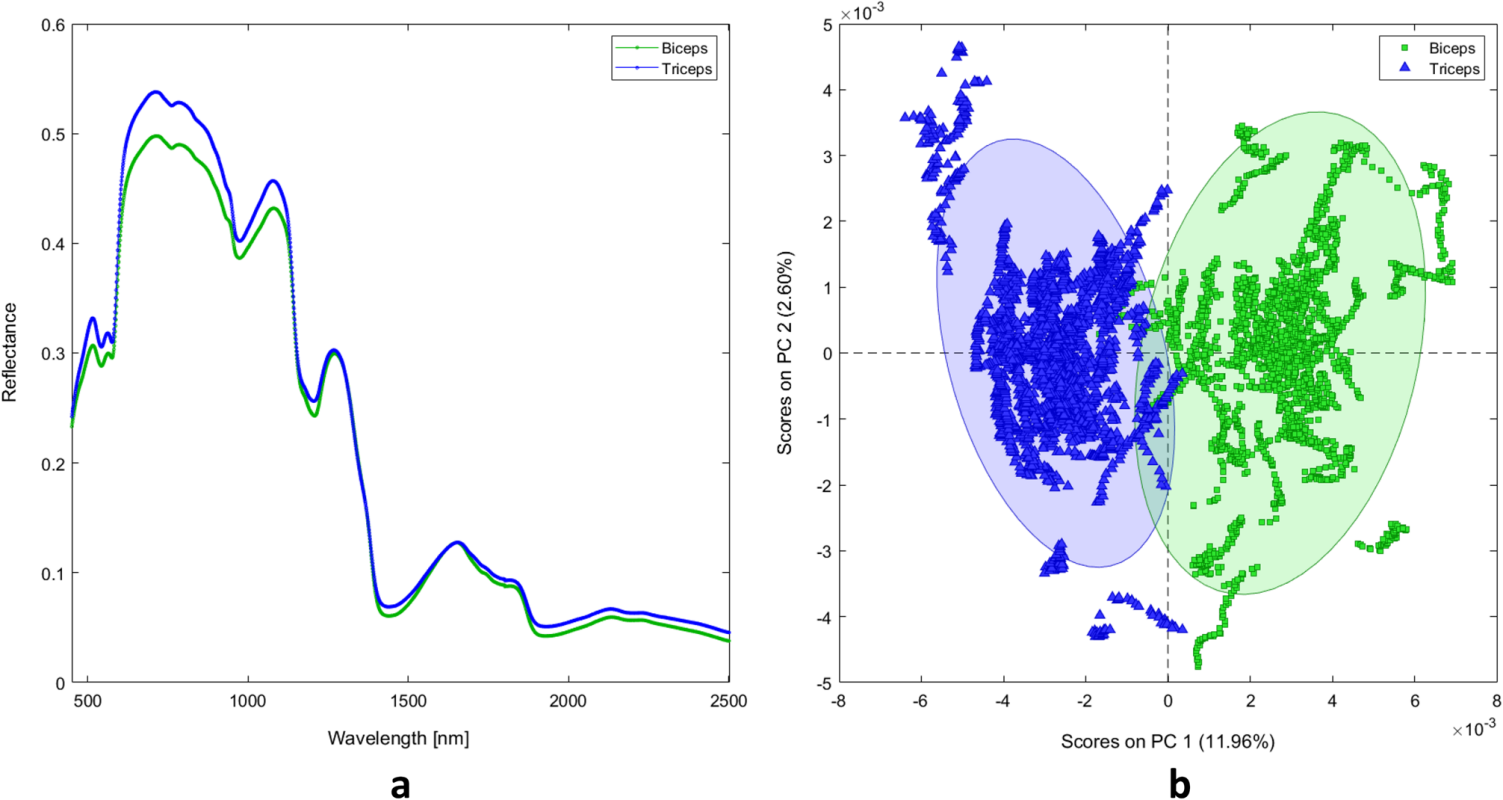
**(a)** Average reflectance spectra of biceps and triceps and **(b)** the related principal component analysis score plot.

Raw mean spectra for the biceps and triceps were collected for each participant (n = 50/muscle/side/patient, n = 50/muscle/normal subjects, total 5400; raw data in the Supplementary Materials File (SMF), supplementary material 2, Figure S1) and grand averages computed for all classes [affected biceps, unaffected biceps, affected triceps, unaffected triceps, normal biceps, normal triceps (Fig. 1); biceps and triceps (Fig. 2a)], together with standard deviation (graphical representations of grand averages and standard deviation for all classes in the SMF, supplementary material 6, Figure S12). Visual inspection of the grand averages revealed that the spectra differ according to the classes. The greatest divergence in the spectra was seen at wavelengths around 760 nm, 970 nm, 1200 nm, and 1440 nm, corresponding to light-absorbing groups^15^.

**Figure 3:**
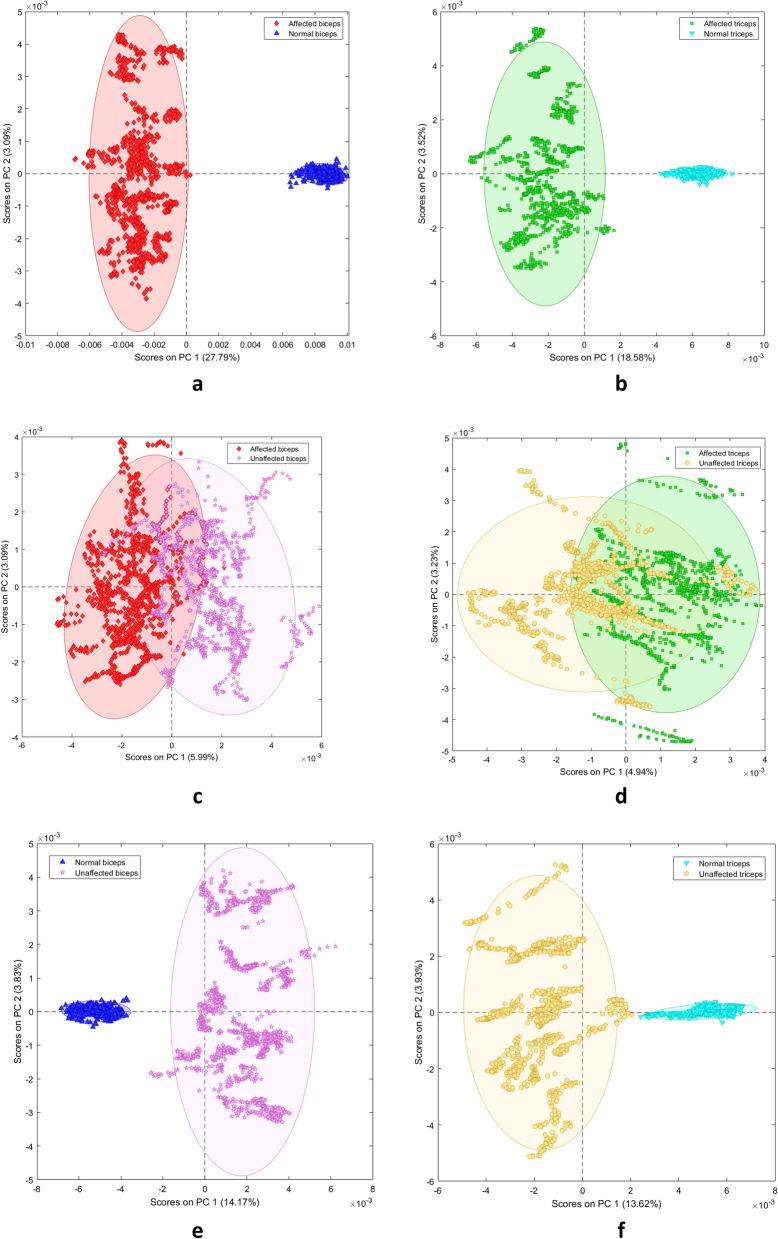
Principal component analysis score plots for **(a)** affected biceps spectra vs. normal biceps spectra; **(b)** affected triceps spectra vs. normal triceps spectra; **(c)** affected biceps spectra vs. unaffected biceps spectra; **(d)** affected triceps spectra vs. unaffected triceps spectra; **(e)** unaffected biceps spectra vs. normal biceps spectra; and **(f)** unaffected triceps spectra vs. normal triceps spectra.

PCA of the main dataset showed that spectra-related scores clustered according to the pairs of classes “biceps/triceps”, “affected/normal”, “affected/unaffected”, and “unaffected/normal”. In addition, scores revealed that PC1 resolved most of the variance between the analyzed classes. The PCA score plots showed that the explained variance (EV) for PC1 for biceps vs. triceps was 11.96% (Fig. 2b), affected vs. normal biceps 27.79% (Fig. 3a), affected vs. normal triceps 18.58% (Fig. 3b), affected vs. unaffected biceps 5.99% (Fig. 3c), affected vs. unaffected triceps 4.94% (Fig. 3d), unaffected vs. normal biceps 14.17% (Fig. 3e), and unaffected vs. normal triceps 13.62% (Fig. 3f).

**Figure 4:**
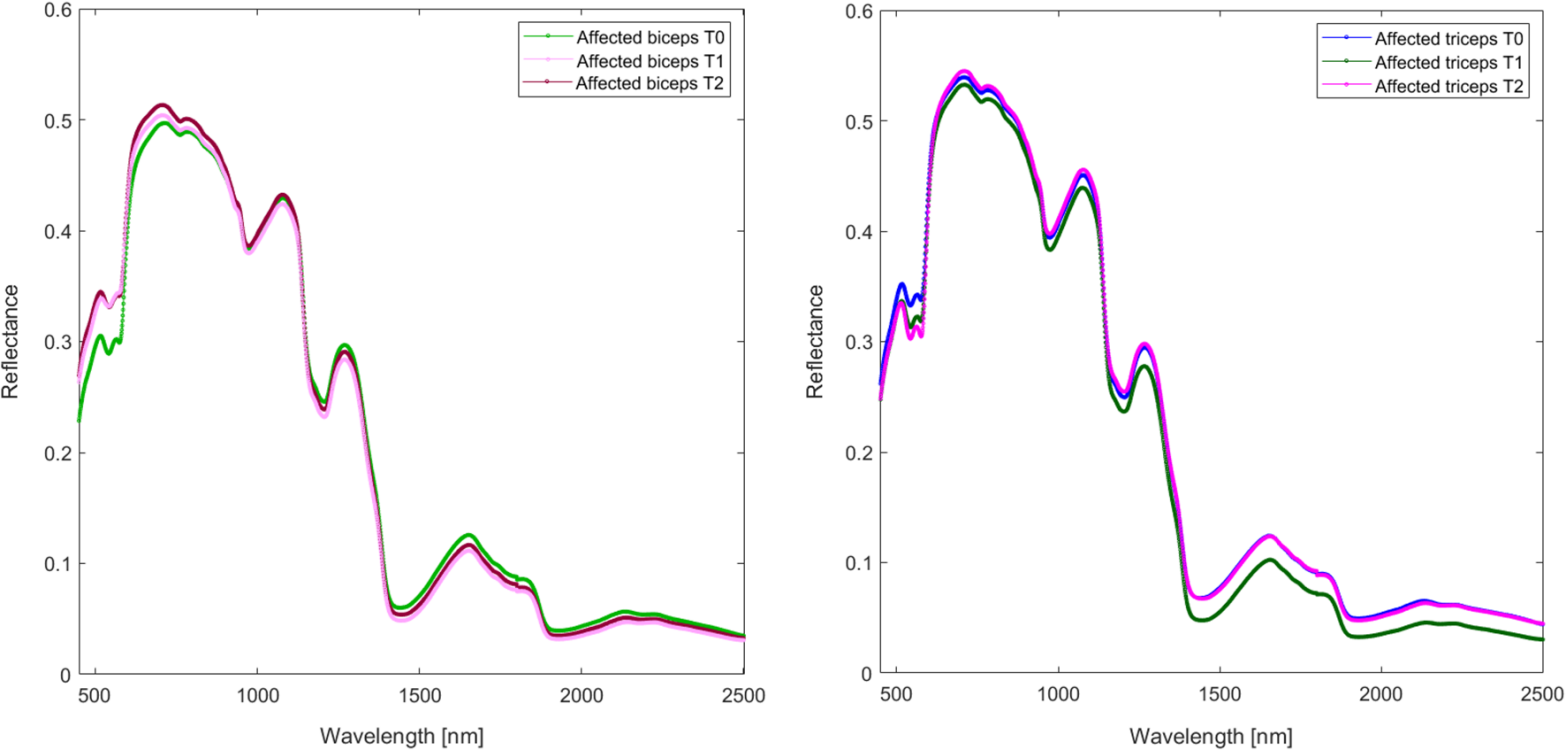
Average reflectance spectra of affected biceps and affected triceps at T0, T1, and T2.

**Figure 5:**
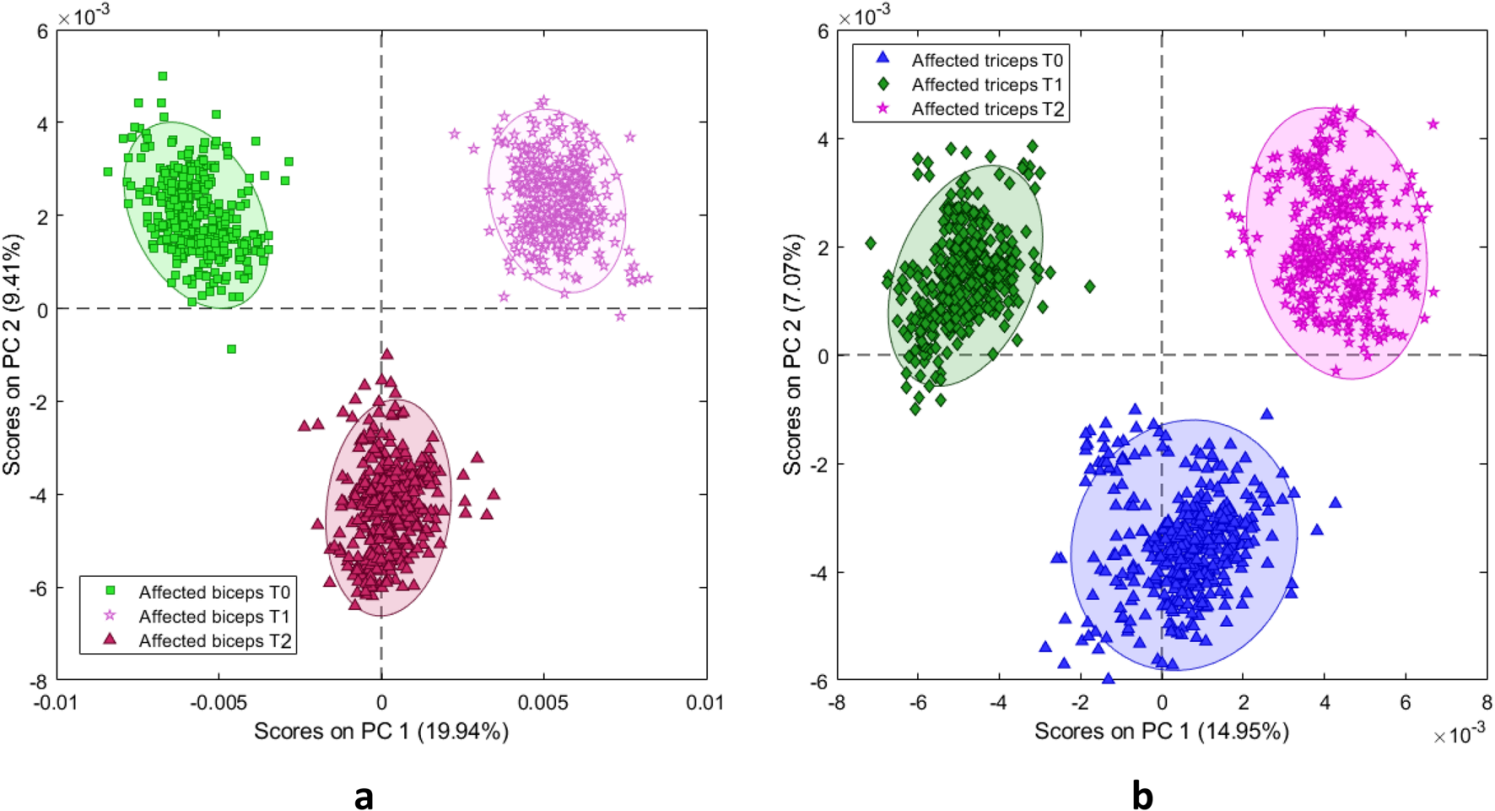
Principal component analysis score plots for **(a)** affected biceps spectra and **(b)** affected triceps spectra at T0 vs. T1 vs. T2.

For spectra acquired from the subgroup of patients treated with incobotulinum toxin A, grand averages were computed for all pairs of classes (affected biceps/affected triceps, T0/T1/T2; Fig. 4). Visual inspection of the grand averages revealed that the spectra differ according to the classes. PCA of the spectra dataset collected from the botulinum-injected patients at T0, T1, and T2 showed that spectra-related scores clustered according to the triads of classes (T0, T1, T2) for both biceps and triceps. PC1 resolved most of the variance between the classes (biceps EV = 19.94% and triceps EV 14.95%; Fig. 5).

Calculations obtained by a Monte Carlo simulation performed on a light propagation model within a multilayered structure based on the magnetic resonance image of the upper arm scanned from one normal participant to the study (SMF supplementary material 3, Figure S2), showed that the light propagates to the muscle both through a fat layer 0.5 cm thick and a fat layer 1.4 cm thick (SMF, supplementary material 3, Figures S4, S5 and S7, S8 and S9).

Among the 23 participants with hypertonia, 12 scored MAS 1 on the biceps and 9 scored MAS 3. On the triceps, 12 scored MAS 1 and 6 scored MAS 3. Patients scoring MAS 2 were the minority, 2 for the biceps and 5 for the triceps. Additional PCA of the spectra dataset collected from the biceps scoring MAS 1, MAS 2, and MAS3 has been performed and presented in the SMF, supplementary material 4. As the Figure S10 shows, the three MAS scores are clearly separated by PC1, that captures about 58% percent of the spectral variance.

**Figure 6:**
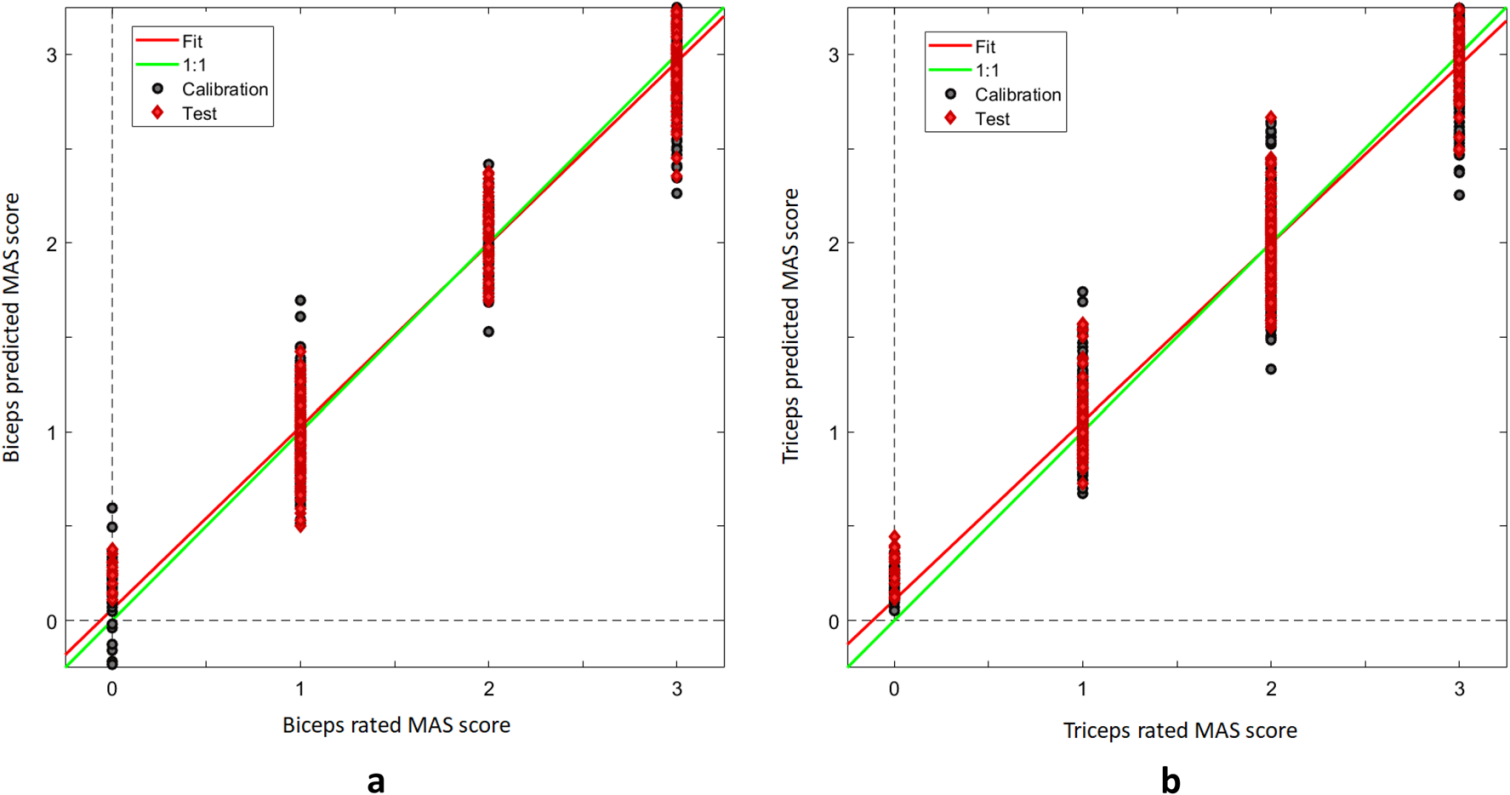
PLS regressions for the clinical measures of hypertonia from reflectance spectra. **(a)** MAS scores in the biceps and **(b)** triceps.

The PLS regression using MAS-biceps scores at T0, T1, and T2 in the subgroup of injected patients reached a coefficient of determination $({R}_{p}^{2})$ of 0.960, RPD of 5, RMSEP of 0.2, and prediction bias of -0.004 (Fig. 6a). The PLS regression using MAS-triceps scores reached an ${R}_{p}^{2}$ of 0.945, RPD of 4.3, RMSEP of 0.2, and prediction bias of 0.014 (Fig. 6b).

PLS-DA classified and predicted the classes of muscle groups. The model’s classification performance was evaluated for each of the pairs of classes “affected/normal”, “affected/unaffected”, and “biceps/triceps” and the triad “T0, T1, T2”. The performance metrics calculated by the confusion matrix evaluating each model of classification are reported in SMF, supplementary material 1, Table S1.

The four separate runs of PCA performed using all 50, only 25, only 10 and only five randomly selected spectra/muscle taken from a previously published dataset^19^ showed that the spectra-related PC scores always clustered according to the pairs of classes “biceps/triceps” independently from the number of spectra subjected to the analysis (SMF, supplementary material 4, Figure S10).

## Discussion

This study of VIS-SWIR reflectance spectroscopy in muscles of patients with post-stroke hemiparesis showed that the optical properties discriminate the muscle (biceps vs. triceps), condition (normal vs. affected vs. unaffected), and effect of botulinum toxin treatment (baseline vs. 30–40 days vs. 110–120 days). Importantly, the present study confirms in a large sample our previous finding, that VIS-SWIR reflectance spectroscopy and chemometric analysis is able to distinguish between biceps and triceps muscles^15^. As we have extensively discussed elsewhere^15^, this is possible due to the identification of muscles’ spectral signatures (i.e., “fingerprints”), which reflect the interaction of light within biological samples, allowing the assessment of a sample’s optical properties in vivo non-invasively. Briefly, photons penetrate tissues and can be reflected or transmitted, and both phenomena are attenuated mainly by absorption and scattering^20^. Organ optical properties are the result of macroscopic differences in the refractive properties of tissues and the microscopic heterogeneities of the refractive indices between extracellular, cellular, and subcellular tissue components. Changes in absorption/reflection/scattering reflect biochemical and/or structural features, which in turn may be specific to anatomy, physiology, or pathology. In this investigation, the selected wavelength interval (450–2500 nm, in which scattering predominates over absorption^21^), the geometry of the optical fiber probe used for radiating and detecting light (35° angle), and the clearance between the fiber-optic probe and the skin surface (direct contact) make the volume probed by near infrared photons extend several centimeters beneath the arm surface and provide spectra that represent average values over such a volume, including skin, fat, vessels, muscles, and possibly bone^15^.

### Factors contributing to the spectral difference between normal elbow muscles

Differences found in the spectra collected from the surface of biceps and triceps did not stem from differences in the corresponding skin probed by VIS-SWIR radiation because of the ineffective arrangement of the factors wavelength/geometry/clearance required for testing the skin. Neither they stemmed from the skinfold thickness, as shown by the calculations of the multilayered light propagation model designed based on a magnetic resonance image of the upper arm scanned from one normal participant to the study (SMF, supplementary material 3).

Because the adipose tissue has no precise oriented structure, and the structures of vessels and bone are invariable when probed from the ventral or dorsal aspect of the arm, the optical properties reflected by the VIS-SWIR spectral fingerprints collected from the biceps and triceps surface can be ascribed to the high degree of spatial organization of the underlying muscle tissue. Due to its ordered structural constitution, the macroscopic arrangement of muscle fibers (i.e., pennation angle, fiber length, cross-sectional area, all determinants of the muscle architecture^22^) can be summarized and substituted by the spectral fingerprints. Distinct muscle architectures can explain most of the differences between biceps and triceps spectra, because the biceps has a parallel architecture^23^, whereas the triceps has a bipennate architecture^24^. Another factor that may contribute to distinguishing the optical properties of muscles is fiber composition, i.e., the relative percentage of slow and fast fibers, as both surface and deep components of the biceps are composed of relatively fewer fast-twitch muscle fibers than corresponding components of the triceps^25,26^. At 90° of elbow flexion, the biceps brachii muscle tendon length is relatively short, whereas the triceps brachii muscle tendon length is relatively long. Because the relationship between muscle tension and length is related to the changes of the structure of the myofibril at the sarcomere level and ultimately to the number of cross-bridges between the filaments^27^, possible distinct passive tension of biceps and triceps muscle–tendon units may be another factor contributing to the spectral difference between normal elbow muscles. This reasoning finds support in the measures taken from specimens including elbow muscles fixed at 90° elbow flexion showing that the optimal fascicle length (i.e., a normalized measure of the number of sarcomeres in series based on the standardization of the muscle fiber length to a standard sarcomere length within a specimen)^28^ of combined biceps heads is longer than that of combined triceps long and lateral heads (13.6 ± 3.7 vs. 10.3 ± 2.5 cm)^29^. Normalized muscle length may therefore be considered a measure of one structural counterpart of the spectral fingerprints that individualize distinct muscles, an especially useful measure because sarcomere length normalization effectively eliminates the joint-angle dependent variation in fiber length^28^.

A further possible contributor to muscle spectral fingerprints is the intramuscular connective tissue, which differs in composition and structure both within and between muscles^30^.

Finally, also when the macroscopic arrangement of muscle fibers is changed—as it happens in biceps and triceps when the elbow angle is changed from 30° (biceps short, triceps long) to 120° (biceps long, triceps short)—VIS-SWIR reflectance spectra still separate the same muscles either placed in an elongated or a shortened position^31^.

### Factors contributing to the spectral difference between affected and normal/unaffected elbow muscles

The present study also found that biceps and triceps spectral fingerprints change according to the condition of the muscle. Many muscle architecture parameters differ in the paretic limbs of stroke patients compared to both contralateral unaffected limbs and normal subjects. In the upper limb, shorter fascicles have been reported^10^, along with smaller total muscle volume and contractile element volume^32^. In the elbow flexors^33^, these changes are supposedly related to mechanisms similar to those operating in muscle immobilization at shortened muscle–tendon lengths, a fundamental muscle adaptation process reported in classic limb immobilization studies in animal models^34^.

Not only does immobilization change the sarcomere length^33,35^, but it also induces loss of serial sarcomeres^36^, making the actual muscle length shorter and the full range of joint motion impossible to achieve. In turn, this leads to reduced extensibility of connective tissue structures, such as the joint capsule and ligaments^5^. When the limb is held at a joint posture that places the muscle at a shortened length, the proportion of collagen to muscle fiber also increases, and this proportional increase occurs well before any loss of serial sarcomeres (i.e., after only 2 days)^37^. In a study in rat muscles, after 3 weeks of immobilization, longitudinally and perpendicularly oriented collagen fiber networks in the endomysium were no longer distinguishable from each other, and considerable changes were found in the intramuscular perimysial connective tissue^38^. These reports are of particular relevance for interpreting the findings of the present study, because patients with subacute hemiparesis were studied along with others exhibiting long-standing hemiparesis. However, to detail the mechanisms by which VIS-SWIR reflectance spectroscopy discloses both early and late post-stroke muscle changes, future studies will have to stratify spectra according to stroke chronicity.

Moreover, UMNS has been shown to be capable of changing muscle fiber composition^39^. This type of muscle adaptation occurs because the histochemical properties of the motor units reflect both the unit function and the order of muscle fiber recruitment in movements^40^. In UMNS, due to a reduction in voluntary power, there is selective disuse of high-threshold phasic motor units and, due to hypertonia^41^, a correspondingly increased use of low threshold motor units^42^. Such an imbalance drives a transformation from phasic to tonic type muscles, leading to atrophy of fast fibers and, in long-standing hemiparesis, hypertrophy of slow fibers^39^.

Measure of hypertonia using MAS^8^ provided considerable variation of scores ranging from mild (score 1) to severe (score 3) hypertonia. We consider hypertonia as one important—although not the only one—contributor to the spectral difference between affected and normal/unaffected elbow muscles. Contribution of hypertonia to spectral fingerprints is indirectly shown in Fig. 5, where the muscles enlightened at T0, T1 and T2—time points when the MAS scores differ—are clearly separated by the PC1, and in Fig. 6, where the MAS scores and the spectral fingerprints prove to be strongly correlated. Direct evidence comes from the analysis of patients’ subgroups of equal MAS scores, showing that spectra separate the muscles scoring MAS 1, 2 or 3 (SMF, supplementary material 4, Figure S10).

All the factors above reasonably contribute to altering the organization of muscles and ultimately change their optical properties, making photonic analysis a sensitive procedure by which to disclose such factors.

### Factors contributing to the spectral difference between antagonist-affected elbow muscles (affected biceps vs. affected triceps)

Spasticity does not abolish the difference in optical properties due to muscle architecture, fiber composition, and intramuscular connective tissue that distinguish the biceps from the triceps. Nevertheless, PCA separated affected biceps and triceps from their corresponding healthy control muscles, indicating that some element of the spectral signature should be ascribed to the differential effect that the UMNS exerts on the upper limb flexors and extensors. An example of such a differential effect is weakness, which affects biceps and triceps differently, as shown by studies measuring the maximal voluntary strength of elbow flexors and extensors^43^. Another example is spastic dystonia, an efferent phenomenon mediated by a tonic supraspinal drive to spinal motor neurons causing spontaneous, involuntary tonic muscle contraction at rest^3^. In the overwhelming majority of patients with post-stroke hemiparesis^44^, by inducing an involuntary coupling of elbow, wrist, and finger flexor muscles, spastic dystonia promotes what is also known as “flexion synergy”, altering the resting posture and bringing the arm into a flexed position throughout the day. Imbalance in elbow activity in favor of flexors leaves the triceps relatively inactive, likely triggering distinct adaptive changes in the two antagonist muscles, i.e., distinct passive tension of biceps and triceps muscle–tendon units. Other factors including the difference in sarcomere length (post-stroke changes induced by shortened position involve one but not the other of the antagonists), and consequently normalized muscle length (longer in healthy biceps than in triceps as reported beforehand)^29^, may contribute to distinguish the spectral fingerprints of affected elbow antagonists as well.

### Factors contributing to the spectral difference between unaffected and normal elbow muscles

A number of studies have shown that the unaffected limb of a stroke patient is altered from normal. The unaffected limb presents exaggerated muscle reflex responses and decreased velocity thresholds^45^, more pronounced defective modulation of EMG activity in flexors than extensors^46^, and larger than normal passive stiffness due to non-contractile muscle elements^47^. Therefore, both neural (i.e., interlimb innervation at the spinal cord level inducing reciprocal inhibition of contralateral muscles) and non-neural (i.e., changes in collagen and muscle tissue) mechanisms induce adaptations in the “good” limb muscles that are revealed by VIS-SWIR reflectance spectroscopy.

Another possible contributor to interlimb muscle differences in patients may be paratonia^48^, a motor disturbance that typically occurs in patients with cognitive impairment but is also found in healthy subjects^49^ and stroke patients (personal unpublished observation). When probed with optical devices, the muscles of demented patients with paratonia exhibit increased levels of advanced glycation end products^50^, and this accumulation has been associated with mechanical stiffness and decreased elasticity in muscle tissue^50,51^.

### Factors contributing to the spectral difference between affected muscles before and after botulinum toxin injection (T0 vs. T1 vs. T2)

Botulinum toxin A is the first-line therapy for focal spasticity^52^, and incobotulinum A is safe and effective for treatment of the upper limb^53^. We found that the spectral signatures of the biceps change after botulinum toxin injection. Obviously, at the time of expected maximum toxin effect (i.e., T1), the muscle is “relaxed” due to chemodenervation, and intuitively its optical properties are changed. PLS regression analysis showed that the MAS scores measured in injected patients highly correlated with the VIS-SWIR spectra (Fig. 6), and PLS models showed that it is possible to predict the MAS scores from the spectrum with very low error. We look with favor at VIS-SWIR spectroscopy and, in future studies, will aim to provide a “photonic quantitation” of muscle relaxation (i.e., “toxin-induced benefit”), evaluated by both clinical scales of hypertonia and instrumental devices (EMG, biomechanical measures).

Interestingly, PCA clearly separates the spectral signatures of biceps collected at T2 from those collected at T0 and T1. This is relevant because neurophysiological techniques disclose the toxin-induced changes in the neural networks at a time similar to T1, but they show normalization of the measurements at a time earlier or similar to T2. Therefore, VIS-SWIR reflectance spectroscopy is able to highlight differences in the current status of the muscle at times when the clinical neurophysiology is no longer sensitive to changes in neural networks.

Interestingly, botulinum toxin not only changed the optical properties of the injected biceps over time, but also did the same on the antagonist, non-injected triceps. This finding can be attributed to the relative changes in muscle length between antagonist elbow muscles (i.e., if the one shortens, the other lengthens) and to indirect, and possibly direct, central effects of botulinum toxin at segmental and suprasegmental levels^54^.

### Methodological considerations and limitations of the study

Because of the proof-of-concept design of the study, patients were not stratified according to stroke chronicity.

No quantitative measurement (e.g., electromyogram) other than a waiting period of at least 60 s was adopted to ensure that the enlightened muscles were relaxed in post-stroke patients. Nevertheless, this time proved enough to allow spastic dystonia to wane in patients with post-stroke spasticity^44^.

Because we aimed to standardize the positions of the probe over the surface of the upper arm, we chose well-known sites, that ideally were “over the muscle belly”. Motor points were selected as the most reliable sites for probe positioning since clinical neurophysiology shows that electrodes positioned over these points pick-up the maximum EMG signal from the muscle. However, cadaver anatomy shows that intimate relationships between tendons and muscle fibers for both biceps and triceps cannot be readily predicted from the surface. The distal biceps tendon flattens into a straplike internal aponeurosis located along the distal centerline of the muscle that spans 34% of the length of biceps brachii long head^55^. The medial head of the triceps has a tendon that lies deep to, and is initially separate from, the tendon shared by the long and lateral heads^56^. The tendons of all three heads insert on the olecranon process of the ulna. Therefore, it cannot be excluded that some tendon component was under the probe. Nevertheless, positioning the probe over the arm surface reproducibly allowed us to explore the underlying tissues with minimal spatial variation among experimental sessions and study participants.

Biological tissues—and organs—are densely packed scattering media. The scattering effect is caused by the refractive index mismatch at the boundaries of different components of cells, such as cell membranes, organelles etc. Photon migration in biological tissue shows strong scattering effects because the inhomogeneities of cellular structures and particle size are larger than wavelengths in the near infrared^57^. Sensitivity of near infrared light to tissue inhomogeneities is therefore informative, because the reflectance measured at detectors takes into accounts contributions from all the components of the sample studied. Directly assessing the specific optical properties of those components would be a reasonable advancement on this experimental design, however it should be emphasized that at this stage of the research, gaining information about the intimate physical chemical composition of normal and post-stroke living muscles could not be—and was not—the aim of the investigation. Rather, we sought to confirm in a highly prevalent clinical condition of muscles secondary to neurological impairment, that VIS-SWIR reflectance spectroscopy can be an objective indicator of the current state of the muscle tissue, that could be measured accurately and observed reproducibly from outside the organ itself. Further studies are warranted to understand in detail the individual factors in muscles that give our method its discriminatory capability.

## Conclusions

The combined application of VIS-SWIR reflectance spectroscopy and chemometrics allows the prediction of chemical constituents in a sample and the use of spectra as surrogate markers of the complex attributes of organic structures. These features were valuable in investigating both affected and unaffected upper limb muscles in patients with post-stroke hemiparesis, revealing that near-infrared-related optical properties actually assess the structure of muscles, reliably distinguishing whether they are flexors or extensors (i.e., the type of muscle, biceps vs. triceps), healthy or diseased (i.e., the condition, normal vs. affected vs. unaffected), and under the effect of botulinum toxin treatment (i.e. having not been treated, having been treated 30 to 40 days or 110 to 120 days prior). The availability of small, simple, cost-effective, quick point-of-care spectroscopic devices renders the photonic evaluation of muscles a useful adjunct to clinical assessment, which may prove invaluable in the assessment and prognosis of hemiparetic muscles affected by stroke. After extended libraries of spectra are collected and classified, VIS-SWIR reflectance spectroscopy combined with MAS scores and other instrumental measurements has the potential to provide a quick, objective, and reliable measure of stroke-induced changes in muscles. In addition, photonic evaluation may allow a more comprehensive, reliable, and reproducible assessment of the effects of different interventions, including physiotherapy and medication, on hemiparetic muscles.

## Materials and methods

### Subjects

Participants in this study were Caucasian southern European individuals enrolled at the Academic Neurology Unit, Terracina (LT), Italy. Normal subjects (n = 8, age 25–87 years; 5 women) were chosen from among laboratory staff and patients’ relatives. Patients (n = 23, age 26–89 years; 8 women) with post-stroke UMNS were chosen from among patients hospitalized after an acute cerebrovascular event and those coming for treatment with botulinum toxin (Table 1). All patients were selected according to the following criteria: (1) clinical presentation of a hemispheric stroke leading to unilateral motor deficit, either subacute (n = 6) or chronic; (2) no previous treatment with botulinum toxin in the 120 days prior; (3) no cognitive impairment (MMSE > 24); and (4) no skin abnormalities or discoloration involving the upper limb. The tone and strength of the elbow muscles were rated according to the Modified Ashworth Scale (MAS)^8^ and the Medical Research Council (MRC) scale, respectively^6^. All participants provided written informed consent before being included in the study, which was approved by the local ethics committee (Comitato Etico Lazio 2, protocol number 0167183/2018). All methods were carried out in accordance with the relevant guidelines and regulations.

**Table 1:** Demographic and anthropometric data for the study participants.

Subject	Group	Age, years	Sex	Side	Lesion	T0 MAS-biceps	T0 MAS-triceps
1	P	41	F	Left	Ischemic	3	2
2	P	85	M	Left	Ischemic	3	3
3	P	65	F	Left	Ischemic	1	2
4	P	54	M	Left	Hemorrhagic	1	3
5	P	74	M	Left	Hemorrhagic	1	1
6	P	75	F	Left	Ischemic	3	3
7	P	42	F	Left	Ischemic	3	3
8	P	72	F	Left	Hemorrhagic	1	1
9	P	65	M	Left	Hemorrhagic	3	2
10	P	25	M	Left	Hemorrhagic	2	2
11	P	68	M	Left	Hemorrhagic	1	1
12	P	48	M	Left	Hemorrhagic	1	1
13	P	55	F	Left	Ischemic	1	1
14	P	87	M	Left	Ischemic	1	1
15	P	51	F	Left	Ischemic	3	1
16	P	89	M	Right	Ischemic	3	3
17	P	73	M	Right	Hemorrhagic	3	3
18	P	52	F	Right	Hemorrhagic	1	1
19	P	66	M	Right	Ischemic	1	1
20	P	59	M	Right	Ischemic	1	1
21	P	56	M	Right	Ischemic	2	1
22	P	71	M	Right	Ischemic	1	1
23	P	59	M	Right	Ischemic	3	2
Mean ± SD	P	62 ± 16	–	–	–	2 ± 1	2 ± 1
24	C	43	M	–	–	–	–
25	C	45	M	–	–	–	–
26	C	33	M	–	–	–	–
27	C	41	F	–	–	–	–
28	C	46	F	–	–	–	–
29	C	54	F	–	–	–	–
30	C	26	F	–	–	–	–
31	C	87	F	–	–	–	–
Mean ± SD	C	47 ± 18	–	–	–	–	–

### Spectra collection and analysis

The portable spectroradiometer system, instrument calibration, in vivo spectra acquisition, spectral data handling, and analysis have been described in detail elsewhere^15^. Briefly, we used a ASD FieldSpec® 4 Standard-Res spectroradiometer (ASD Inc., Boulder, CO, USA)^58^ that works in the spectral range of 350–2500 nm. This instrument has different, separate holographic diffraction gratings with three separate detectors: a VIS detector (350–1000 nm), a SWIR1 detector (1001–1800 nm), and a SWIR2 detector (1801–2500 nm). The contact probe consists of a halogen bulb light source with a 12° light source angle, 35° measurement angle, and a spot size of 10 mm. The native software for the ASD instrument, called RS3, was used for device calibration and data acquisition.

The calibration procedure included dark acquisition and measurement of white reference material (Spectralon white reference standard from LabSphere™) in .asd data files that were imported into the MATLAB® (MATLAB R2019b, ver. 8.4; The Mathworks, Inc.) environment using an *ad hoc* script. Imported data files were analyzed using the Eigenvector Research, Inc., PLS_toolbox (ver. 8.2.1)^59^. Data were stored in dataset objects, and classes were set.

VIS-SWIR reflectance spectra were acquired from biceps and triceps after cleaning both the probe and the skin contact zone with disposable skin cleaning wipes. The instrument contact probe was placed on the subject’s skin and the spectroradiometer controlled from a remote laptop. The position of the contact probe for spectra collection was standardized according to the positions of muscle motor points^60^. For the biceps, the motor point is just distal to the midportion of the muscle. At this level (below to the midshaft of the humerus), the two biceps heads originating from the scapular bicipitolabral complex (long head) and the tip of the scapular coracoid process (short head) have converged to form a common muscle belly. For the triceps, the muscle fibers in the three heads converge towards a large aponeurosis which begins near the middle of the muscle. The end plates are arranged in a long longitudinally oriented oval band, with the crown of the oval situated proximally^61,62^. The position of this oval crown is approximately at the half of the posterior aspect of the upper arm.

The limb was passively placed and maintained in the requested position (i.e., elbow angle approximately at 90°, neutral forearm rotation) for at least 60 s before starting the acquisition, a time considered sufficient to allow spastic dystonia to wane in patients with post-stroke spasticity^44^. Spectra were then acquired only when both the examiner (through palpation) and the patient (by subjective feeling) concorded that the muscle was relaxed. When the acquisition started, the patients were instructed to maintain the muscles “relaxed” for the time needed for acquisition (usually 60 s). Patients were asked to alert experimenters if they felt any uncomfortable sensation during the procedure.

With the segment fully supported and the limb held in the required fixed posture, 50 spectra were acquired from biceps and triceps on the affected and unaffected sides in patients and a randomly chosen side in healthy controls. One hundred spectra/normal subject and 200 spectra/patient were recorded for a total of 5400 spectra (main dataset, 23 patients and 8 normal subjects). The time needed to acquire 50 spectra/muscle was approximately 60 s, for a total duration per participant of approximately 2 to 4 min.

In a subgroup of 8 chronic patients (Table 2; age 25–85 years, 3 women), spectra acquisition was repeated at different times: T0 (baseline, i.e., before intramuscular injection of botulinum toxin and at least 120 days after the last injection), T1 (30 to 40 days after injection of 50 U incobotulinumtoxin A in the biceps under ultrasound guidance at T0), and T2 (110 to 120 days after injection). A total of 2400 spectra were included in the repeated measures dataset.

**Table 2:** Subgroup of patients injected with incobotulinum A toxin in biceps.

Subject	Age, years	Sex	T0 MAS-biceps	T0 MAS-triceps	T1 MAS-biceps	T1 MAS-triceps	T2 MAS-biceps	T2 MAS-triceps
1	41	F	3	2	1	3	1	3
2	85	M	3	3	1	2	3	2
3	54	M	1	3	0	0	3	2
4	42	F	3	3	1	1	2	3
5	65	M	3	2	1	2	3	1
6	25	M	2	2	1	1	2	2
7	73	M	3	3	1	1	2	2
8	59	M	3	2	1	1	1	2

Chemometric analysis was performed on the main dataset and the repeated measures dataset in the spectral range 450–2500 nm, starting with a select combination of preprocessing steps to remove physical phenomena and optimize the multivariate analysis: extended multiplicative scatter correction (EMSC), generalized least square—weighting (GLS-W) on classes (with α = 0.002, to increase the filtering effect), and mean center (MC) algorithms. Reflectance spectra data were subjected to principal component analysis (PCA), a mathematical procedure designed to resolve sets of data into orthogonal components with linear combinations that approximate the original data to any desired degree of accuracy^63^. Principal components (PCs) were chosen by exploring the eigenvalues plot; outliers were identified and excluded by exploring Hotelling’s T^2^ vs. Q residuals plots.

Seven PCAs were performed on the main dataset according to the following pairs of classes:

- biceps (n = 2700) vs. triceps (n = 2700);
- affected biceps (n = 1150) vs. normal biceps (n = 400);
- affected biceps (n = 1150) vs. unaffected biceps (n = 1150);
- affected triceps (n = 1150) vs. normal triceps (n = 400);
- affected triceps (n = 1150) vs. unaffected triceps (n = 1150);
- unaffected biceps (n = 1150) vs. normal biceps (n = 400);
- unaffected triceps (n = 1150) vs. normal triceps (n = 400).
  Two PCAs were performed on the repeated measures dataset to explore spectra collected at the three time points according to the following triads of classes:
- affected biceps T0 vs. affected biceps at T1 vs. affected biceps at T2 (n = 1200);
- affected triceps T0 vs. affected triceps at T1 vs. affected triceps at T2 (n = 1200).
  Partial least squares (PLS) regression^64^ modelling was used to evaluate the correlation between spectra and clinical measures of hypertonia (MAS-B and MAS-T) in repeated measures datasets. Using the Kennard–Stone (K–S) algorithm, 70% of the spectral samples were randomly selected to build up a calibration set; the remaining 30% were used for validation. Cross-validation was performed using Venetian blinds in each model to tune the calibrations. Distinct latent variables were chosen for the PLS of MAS-biceps and MAS-triceps. The goodness of fit of the spectral data in the regression model was assessed with the coefficient of determination R^2^, the ratio of standard error of performance to standard deviation (RPD), the root mean square error (RMSE), and the bias.
  To classify and predict the classes of muscle groups, we used PLS discriminant analysis (PLS-DA)^65^, a pattern recognition method that explores the predictive ability between predictor and response variables. Calibration and validation sets were built using the K–S algorithm. PLS-DA models were subjected to the Venetian blinds cross-validation method. To evaluate the performance of the classification model, we used the confusion matrix with commonly used performance metrics: precision, accuracy, misclassification error, sensitivity, and specificity^66^. PLS-DA models were subjected to the Venetian blinds cross-validation method^15^.
  Nine classification models were established for recognizing:
- biceps (n = 2700) vs. triceps (n = 2700);
- affected biceps (n = 1150) vs. normal biceps (n = 400);
- affected biceps (n = 1150) vs. unaffected biceps (n = 1150);
- affected triceps (n = 1150) vs. normal triceps (n = 400);
- affected triceps (n = 1150) vs. unaffected triceps (n = 1150);
- unaffected biceps (n = 1150) vs. normal biceps (n = 400);
- unaffected triceps (n = 1150) vs. normal triceps (n = 400);
- affected biceps T0 vs. affected biceps at T1 vs. affected biceps at T2 (n = 1200);
- affected triceps T0 vs. affected triceps at T1 vs. affected triceps at T2 (n = 1200).

To demonstrate that the distinct skinfold thickness does not prevent the light from our probe to reach the muscle layer, we designed a light propagation model within a multilayered structure based on a magnetic resonance image of the upper arm scanned from one normal participant to the study (SMF, supplementary material 3, Figure S2), who gave informed consent both for MRI scanning and to publish the image in an online open-access publication. The model was used to perform a Monte Carlo simulation that adopted the layers thickness as measured on the magnetic resonance imaging scan, and the reduced scattering and absorption coefficients for each layer as reported in the literature of the field^67^ (SMF supplementary material 3, tables S2, S3, S4, figures S3 and S6).

To test whether the number of spectra acquisitions (50, intra-individual data) influences the results of the chemometric analysis, in the SMF, supplementary material 5, by using our previously published dataset designed for the purpose of making supplementary studies^19^, we have replicated four times the same statistical analysis, the first time using all acquired 50 spectra/muscle, the second using only 25, the third using only 10, and the fourth using only 5 randomly selected spectra/muscle.

## Supplementary Information

Supplementary Information.

## Data Availability

The datasets generated during and/or analyzed during the current study are available from the corresponding author on reasonable request.
