# Supplementary material for: In vivo non-invasive near-infrared spectroscopy distinguishes normal, post-stroke, and botulinum toxin treated human muscles
Source: Sci Rep. 2021 Sep 3;11:17631. doi: 10.1038/s41598-021-96547-6 (PMC8417034; doi:10.1038/s41598-021-96547-6)
Supplement: Supplementary file 1 — Supplementary Information. [file 41598_2021_96547_MOESM1_ESM.pdf]

Supplementary materials

Supplementary material 1

Table S1. Performance metrics in calibration, cross-validation, and validation using PLS-DA models for discriminating pairs or triads of classes.

Biceps and Triceps spectra at T0

	Class	*Sensitivity*	*Specificity*	*Misclassification Error*	*Precision*	*Accuracy*
Calibration	Biceps	0.990	1.000	0.005	1.000	0.995
	Triceps	1.000	0.990	0.005	0.990	0.995
Cross-validation	Biceps	0.990	1.000	0.005	1.000	0.995
	Triceps	1.000	0.990	0.005	0.990	0.995
Validation	Biceps	0.995	1.000	0.002	1.000	0.998
	Triceps	1.000	0.995	0.002	0.995	0.998

Affected biceps spectra at T0 and normal biceps spectra

	Class	*Sensitivity*	*Specificity*	*Misclassification Error*	*Precision*	*Accuracy*
Calibration	Affected biceps	1.000	1.000	0.000	1.000	1.000
	Normal biceps	1.000	1.000	0.000	1.000	1.000
Cross-validation	Affected biceps	1.000	1.000	0.000	1.000	1.000
	Normal biceps	1.000	1.000	0.000	1.000	1.000
Validation	Affected biceps	1.000	1.000	0.000	1.000	1.000
	Normal biceps	1.000	1.000	0.000	1.000	1.000

Affected triceps spectra at T0 and normal triceps spectra

	Class	*Sensitivity*	*Specificity*	*Misclassification Error*	*Precision*	*Accuracy*
Calibration	Affected triceps	1.000	1.000	0.000	1.000	1.000
	Normal triceps	1.000	1.000	0.000	1.000	1.000
Cross-validation	Affected triceps	1.000	1.000	0.000	1.000	1.000
	Normal triceps	1.000	1.000	0.000	1.000	1.000
Validation	Affected triceps	1.000	1.000	0.000	1.000	1.000
	Normal triceps	1.000	1.000	0.000	1.000	1.000

Affected and unaffected biceps spectra at T0

	Class	Sensitivity	Specificity	Misclassification Error	Precision	Accuracy
Calibration	Affected biceps	0.982	0.988	0.015	0.989	0.985
	Unaffected biceps	0.988	0.982	0.015	0.981	0.985
Cross-validation	Affected biceps	0.976	0.986	0.019	0.987	0.981
	Unaffected biceps	0.986	0.976	0.019	0.975	0.981
Validation	Affected biceps	0.835	0.866	0.148	0.841	0.852
	Unaffected biceps	0.866	0.835	0.148	0.862	0.852

Affected and unaffected triceps spectra at T0

	Class	Sensitivity	Specificity	Misclassification Error	Precision	Accuracy
Calibration	Affected triceps	0.971	0.968	0.030	0.970	0.970
	Unaffected triceps	0.968	0.971	0.030	0.969	0.970
Cross-validation	Affected triceps	0.965	0.966	0.034	0.969	0.966
	Unaffected triceps	0.966	0.965	0.034	0.963	0.966
Validation	Affected triceps	0.978	0.941	0.042	0.933	0.958
	Unaffected triceps	0.941	0.978	0.042	0.981	0.958

Normal and unaffected biceps spectra at T0

	Class	Sensitivity	Specificity	Misclassification Error	Precision	Accuracy
Calibration	Normal biceps	1.000	1.000	0.000	1.000	1.000
	Unaffected biceps	1.000	1.000	0.000	1.000	1.000
Cross-validation	Normal biceps	1.000	1.000	0.000	1.000	1.000
	Unaffected biceps	1.000	1.000	0.000	1.000	1.000
Validation	Normal biceps	1.000	1.000	0.000	1.000	1.000
	Unaffected biceps	1.000	1.000	0.000	1.000	1.000

Normal and unaffected triceps spectra at T0

	Class	Sensitivity	Specificity	Misclassification Error	Precision	Accuracy
Calibration	Normal triceps	1.000	1.000	0.000	1.000	1.000
	Unaffected triceps	1.000	1.000	0.000	1.000	1.000
Cross-validation	Normal triceps	1.000	1.000	0.000	1.000	1.000
	Unaffected triceps	1.000	1.000	0.000	1.000	1.000

Validation	Normal triceps	1.000	1.000	0.000	1.000	1.000
	Unaffected triceps	1.000	1.000	0.000	1.000	1.000

Affected biceps spectra at T0, T1, and T2

	Class	*Sensitivity*	*Specificity*	*Misclassification Error*	*Precision*	*Accuracy*
	Affected biceps T0	1.000	1.000	0.000	1.000	1.000
Calibration	Affected biceps T1	1.000	1.000	0.000	1.000	1.000
	Affected biceps T2	1.000	1.000	0.000	1.000	1.000
	Affected biceps T0	1.000	1.000	0.000	1.000	1.000
Cross-validation	Affected biceps T1	1.000	1.000	0.000	1.000	1.000
	Affected biceps T2	1.000	1.000	0.000	1.000	1.000
	Affected biceps T0	1.000	1.000	0.000	1.000	1.000
Validation	Affected biceps T1	1.000	1.000	0.000	1.000	1.000
	Affected biceps T2	1.000	1.000	0.000	1.000	1.000

Affected triceps spectra at T0, T1, and T2

	Class	*Sensitivity*	*Specificity*	*Misclassification Error*	*Precision*	*Accuracy*
	Affected triceps T0	1.000	1.000	0.000	1.000	1.000
Calibration	Affected triceps T1	1.000	1.000	0.000	1.000	1.000
	Affected triceps T2	1.000	1.000	0.000	1.000	1.000
	Affected triceps T0	1.000	1.000	0.000	1.000	1.000
Cross-validation	Affected triceps T1	1.000	1.000	0.000	1.000	1.000
	Affected triceps T2	1.000	1.000	0.000	1.000	1.000
	Affected triceps T0	1.000	1.000	0.000	1.000	1.000
Validation	Affected triceps T1	1.000	1.000	0.000	1.000	1.000
	Affected triceps T2	1.000	1.000	0.000	1.000	1.000

Supplementary material 2: single spectra

All single reflectance spectra from normal biceps, affected biceps, unaffected biceps, normal triceps, affected triceps, and unaffected triceps of the main dataset are reported in Fig. S1.

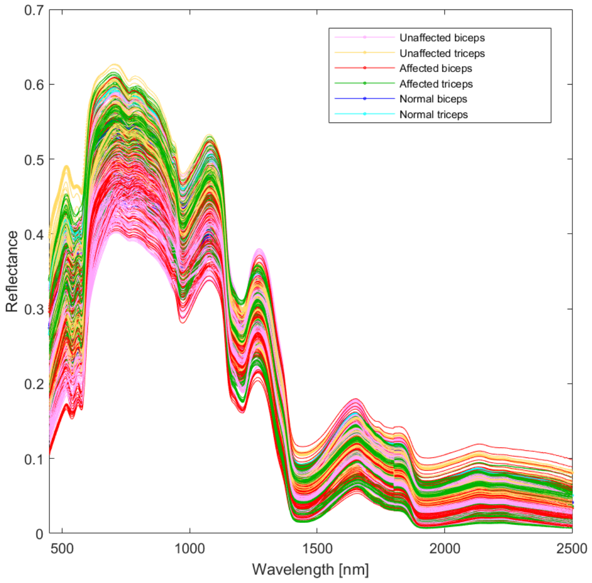

Figure S1. Single raw reflectance spectra acquired from normal biceps, affected biceps, unaffected biceps, normal triceps, affected triceps, and unaffected triceps.

Supplementary material 3: skinfold thickness

2.1 Monte Carlo Simulation: photon migration in the ventral and dorsal sides of an arm.
To demonstrate that distinct skinfold thickness does not prevent the light from our probe to reach the muscle layer, we performed a light propagation simulation within a multi-layered structure designed based on an MRI scan (Fig. S2) of the upper arm taken from one normal participant to the study, who gave informed consent both for MRI scanning and to publish the image in an online open-access publication.

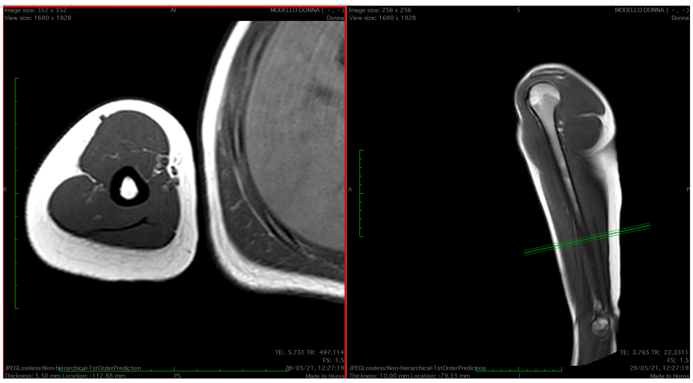

Figure S2. MRI scan of the upper arm taken from one normal participant to the study.

The Monte Carlo simulation was used for modelling light propagation in a multi-layered 3D voxel space (5 cm x 5 cm x 5 cm).
Two simulations were performed: the first representing the ventral aspect of an arm and the second the dorsal side of an arm. The simulations were performed in MATLAB environment by using the tool from Marti et al. 2018.
The optical properties of the single layer were taken from previous studies (Bashkatov et al. 2011; Jacques 2013; Manrique, 2018).
The models considered a radial-factorizable beam with a gaussian distribution for simulating the ASD contact probe with a 12° light source angle, a 35° measurement angle, and a spot size of 10 mm.
The simulations were performed using the optical coefficients at 1069 nm, as reported in Table S1.

Table S2. Optical properties of multi-layered tissue at λ=1069 nm used in the Monte Carlo simulations.
μ_a=coefficient of absorption, μ_s = scattering coefficient, g = anisotropy and n = refractive index.

	μ_a (cm^-1)	μ_s (cm^-1)	g	n
Epidermis	1.71	187.09	0.9	1.3
Dermis	0.08	87.94	0.9	1.3
Fat	0.06	32.99	0.9	1.3
Muscle	0.12	32.99	0.9	1.3
Bone	0.04	140.32	0.9	1.3

2.2 Monte Carlo Simulation: ventral aspect of an arm (biceps).

The geometry illustration of the model is shown in Fig. S3, while the layers thickness of the dorsal aspect of the arm evaluated from the MRI scan are reported in Table S3.

The simulation duration was about 0.200 min. About 7.89e+05 photons were simulated at a rate of 3.94e+06 photons per minute.

About 61% of incident light hits the cuboid boundaries, while 39% of incident light was absorbed within the cuboid.

The graphical illustrations of the simulation are shown in Fig. S4, while the normalized fluence rate vs the penetration depth is shown in Fig. S5.

Table S3. Layers thickness of the ventral aspect of the arm.

	Thickness (cm)
Epidermis	0.06
Dermis	0.20
Fat	0.48
Muscle	2.70

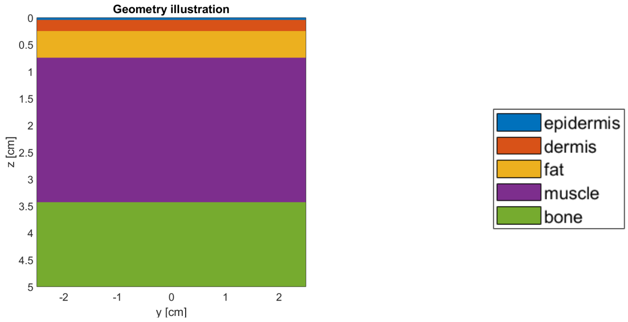

Figure S3. Z-Y geometry illustration of the voxel representing the ventral aspect of an arm.

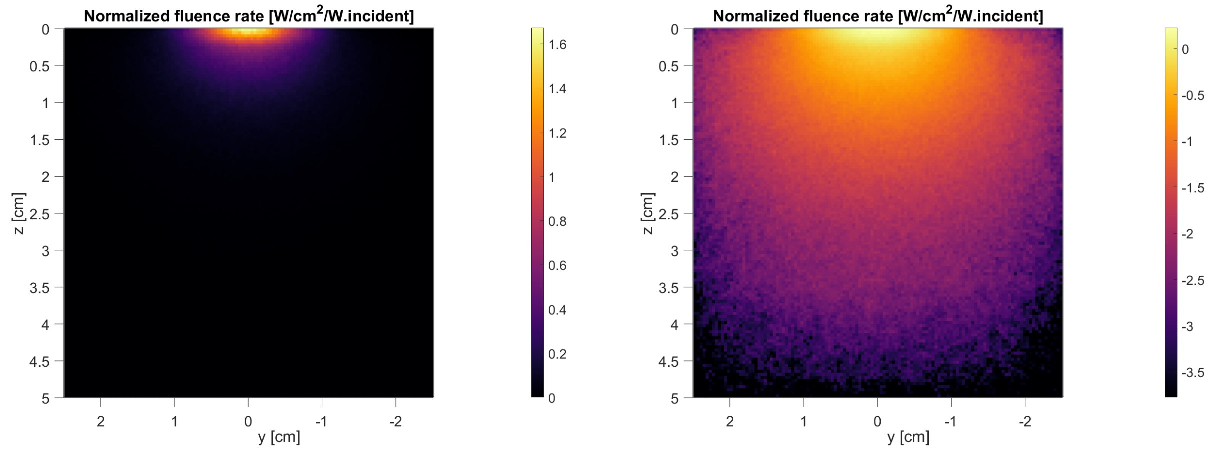

a

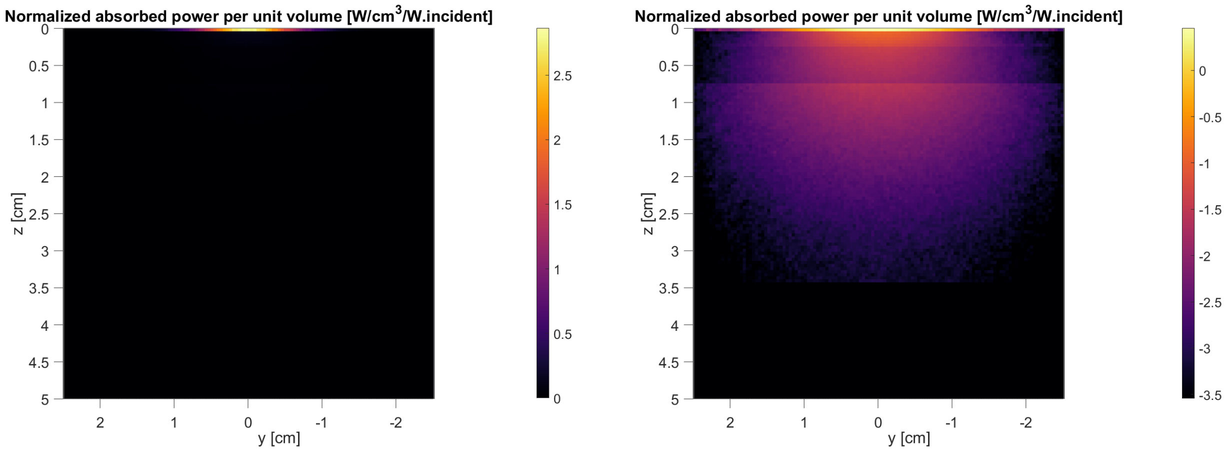

b

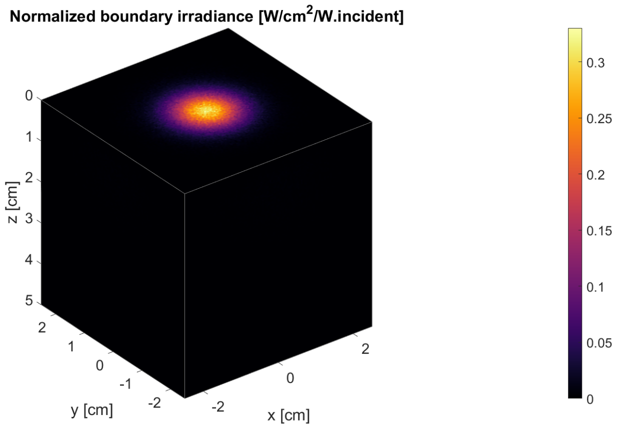

c

Figure S4. Monte Carlo simulation results of light propagation within the ventral aspect of an arm: Z-Y representation of the normalized fluence rate and its corresponding logarithmic representation (a); Z-Y representation of the normalized absorbed unit volume and its corresponding logarithmic representation (b); cuboid representation of the normalized boundary irradiance (c).

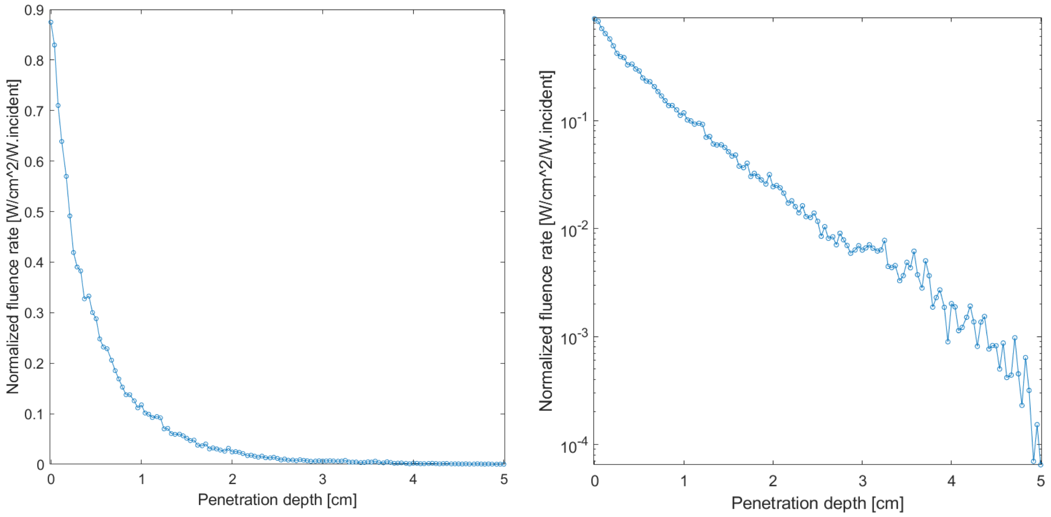

Figure S5. Normalized fluence rate vs. penetration depth within the voxel representing the ventral aspect of an arm (for X=0 and Y=0) (left panel) and the corresponding semi-log representation (right panel).

2.3 Monte Carlo Simulation: dorsal aspect of an arm (triceps).

The geometry illustration of the model is shown in Fig. S6, while the layers thickness of the dorsal aspect of the arm evaluated from the MRI scan are reported in Table S4.

The simulation duration was about 0.200 min. About 77.08e+05 photons were simulated at a rate of 3.54e+06 photons per minute.

About 62.7% of incident light hits the cuboid boundaries, while 37.3% of incident light was absorbed within the cuboid.

The graphical illustrations of the simulation are shown in Fig. S7, while the normalized fluence rate vs. the penetration depth is shown in Fig. S8.

Table S4. Layers thickness of the dorsal aspect of the arm.

	Thickness (cm)
Epidermis	0.06
Dermis	0.20
Fat	1.40
Muscle	1.10

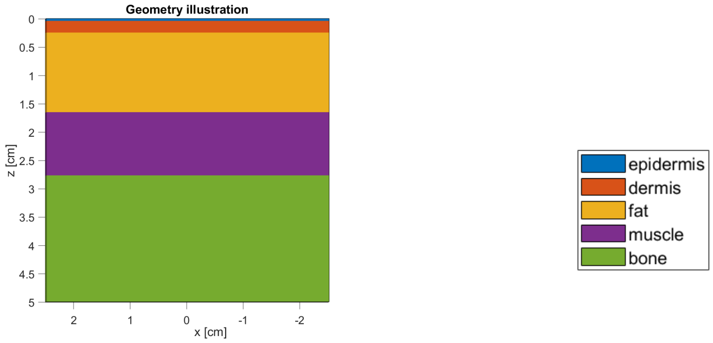

Figure S6. Z-X geometry illustration of the voxel representing the ventral aspect of an arm.

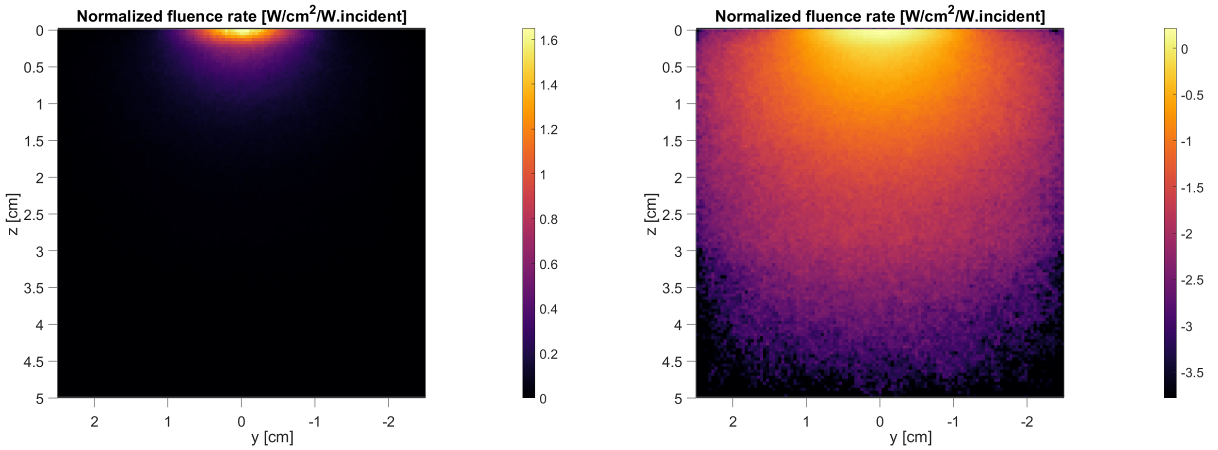

a

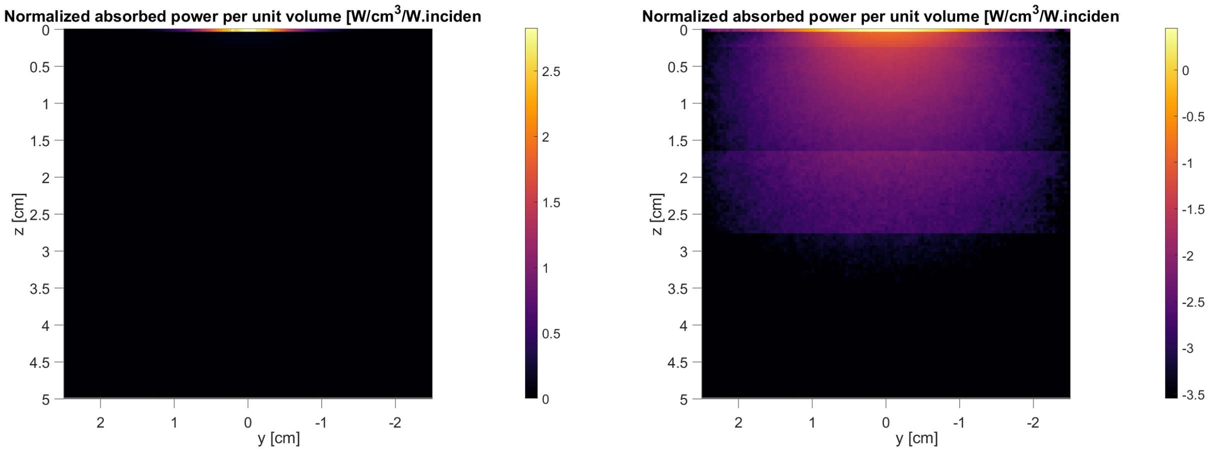

b

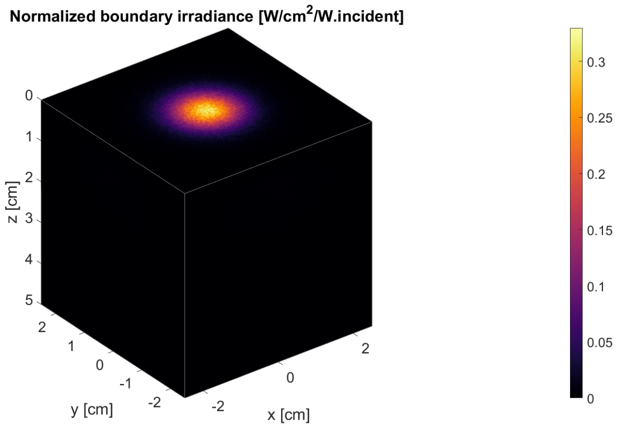

c

Figure S7. Monte Carlo simulation results of light propagation within the dorsal aspect of an arm: Z-Y representation of the normalized fluence rate and its corresponding logarithmic representation (a); Z-Y representation of the normalized absorbed unit volume and its corresponding logarithmic representation (b); cuboid representation of the normalized boundary irradiance (c).

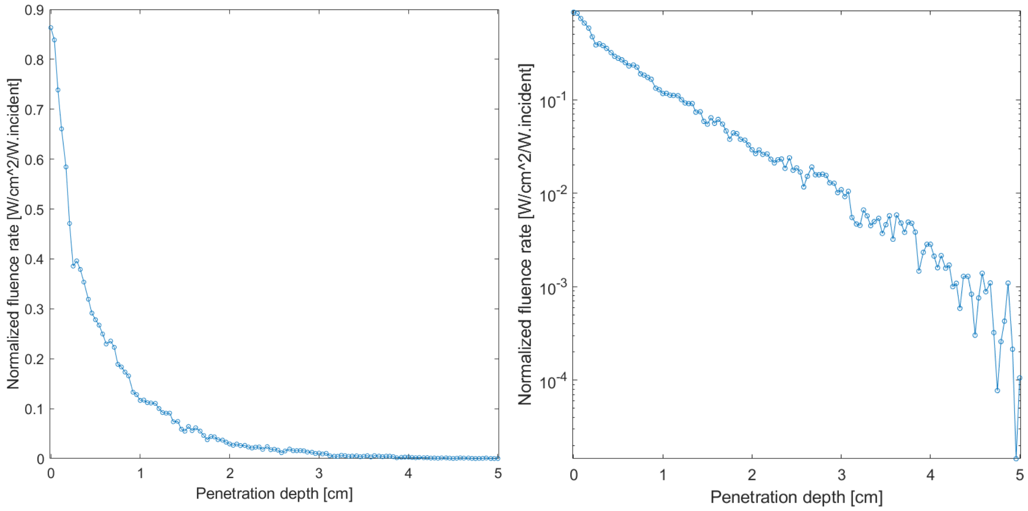

Figure S8. Normalized fluence rate vs. penetration depth within the voxel representing the dorsal aspect of an arm (for X=0 and Y=0) (left panel) and the corresponding semi-log representation (right panel).

2.4 Monte Carlo Simulation: dorsal and ventral aspect of an arm.

The comparison of the normalized fluence rate vs. penetration depth for the dorsal and the ventral aspect of an arm models is shown in Fig. S9.

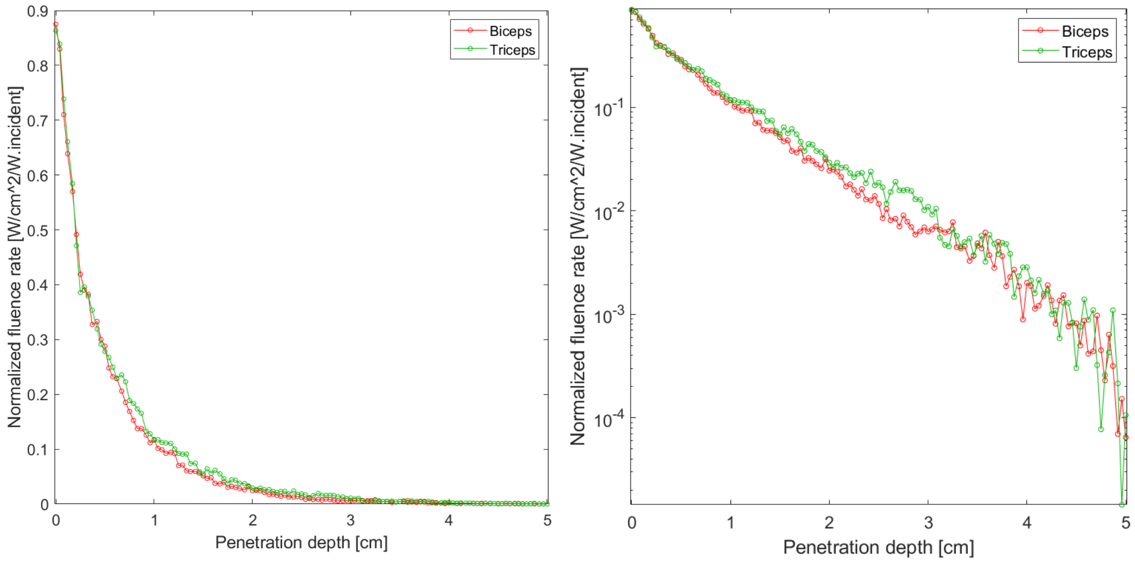

Figure S9. Normalized fluence rate vs. penetration depth for the dorsal and the ventral aspect of an arm (for X=0 and Y=0) (left panel) and the corresponding semi-log representation (right panel).

2.5 Supplementary material 3 References

- Bashkatov AN, Genina EA, Tuchin VV (2011). "Optical properties of skin, subcutaneous, and muscle tissues: a review." Journal of Innovative Optical Health Sciences 4, no. 01: 9-38.
- Jacques SL. (2013). "Optical properties of biological tissues: a review." Physics in Medicine & Biology 58, no. 11: R37.
- Marti D, Aasbjerg RN, Andersen PE, Hansen AK (2018). "MCmatlab: an open-source, user-friendly, MATLAB-integrated three-dimensional Monte Carlo light transport solver with heat diffusion and tissue damage," J. Biomed. Opt.
- Niwayama M, Yamashita Y (2013). Photon migration in tissue. In Application of Near Infrared Spectroscopy in Biomedicine (pp. 21-35). Springer, Boston, MA.
- Rivera-Manrique SI, Brio-Perez M, Trejo-Sanchez JA, Offerhaus HL, Ek-Ek JR, Alvarez Chavez JA (2018). "Thermal Diffusion and Specular Reflection, Monte Carlo-based Study on Human Skin via Pulsed Fiber Laser Energy." In XVI Symposium of Mexican Students and Studies in the UK 2018.

Supplementary material 4: subgroups of equal MAS scores

Among the 23 participants, 12 had MAS 1 on the biceps and 9 had MAS 3. On the triceps, 12 had MAS 1 and 6 had MAS 3. Patients with MAS 2 were the minority, 2 on the biceps and 5 on the triceps. This distribution allows to compare patients with mild hypertonia (MAS 1), moderate (MAS 2) or severe hypertonia (MAS 3).
Therefore, we performed PCA of the T0 spectra acquired from biceps scoring MAS values of 1, 2 and 3. As the Fig. S10 shows, the three MAS score are clearly separated by the first PC that captures about 58% percent of the spectral variance.

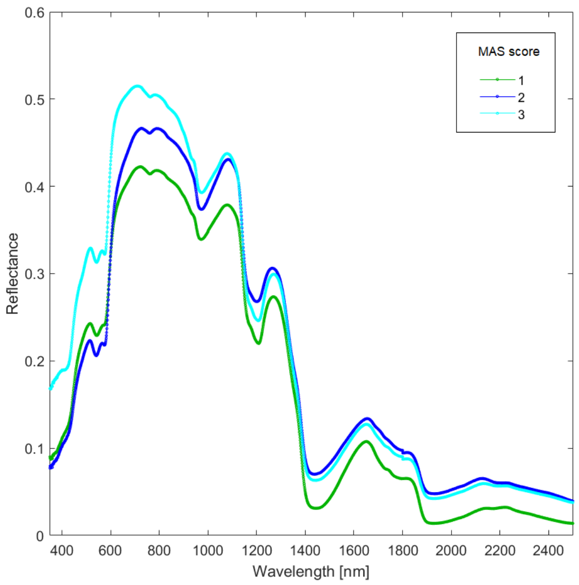

a

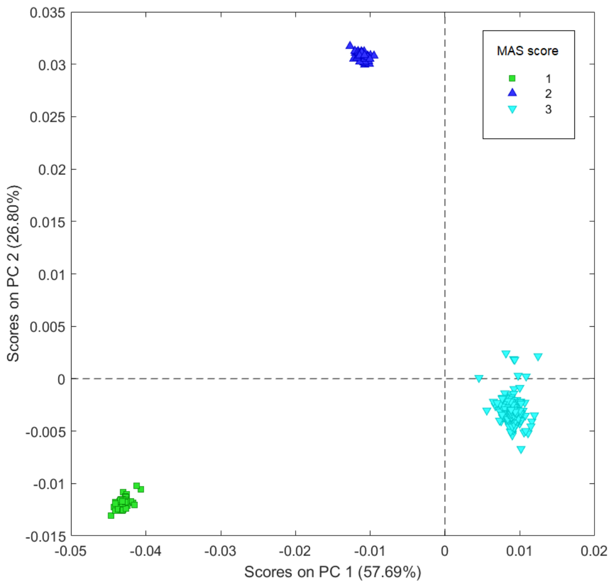

b

Figure S10. Reflectance spectra of biceps averaged according to MAS scores (a) and the related principal component analysis score plot (b).

Supplementary material 5: inter-individual and intra-individual data

To test whether the strength of chemometric analysis changes with the number of spectra acquisitions (intra-individual data), in the supplementary material 4, by using our previously published dataset (Currà et al. 2020, designed for the purpose), we have replicated four times the same statistical analysis, the first time using all acquired 50 spectra/muscle, the second 25, the third 10, and the fourth 5 randomly selected spectra/muscle (Figure S11).

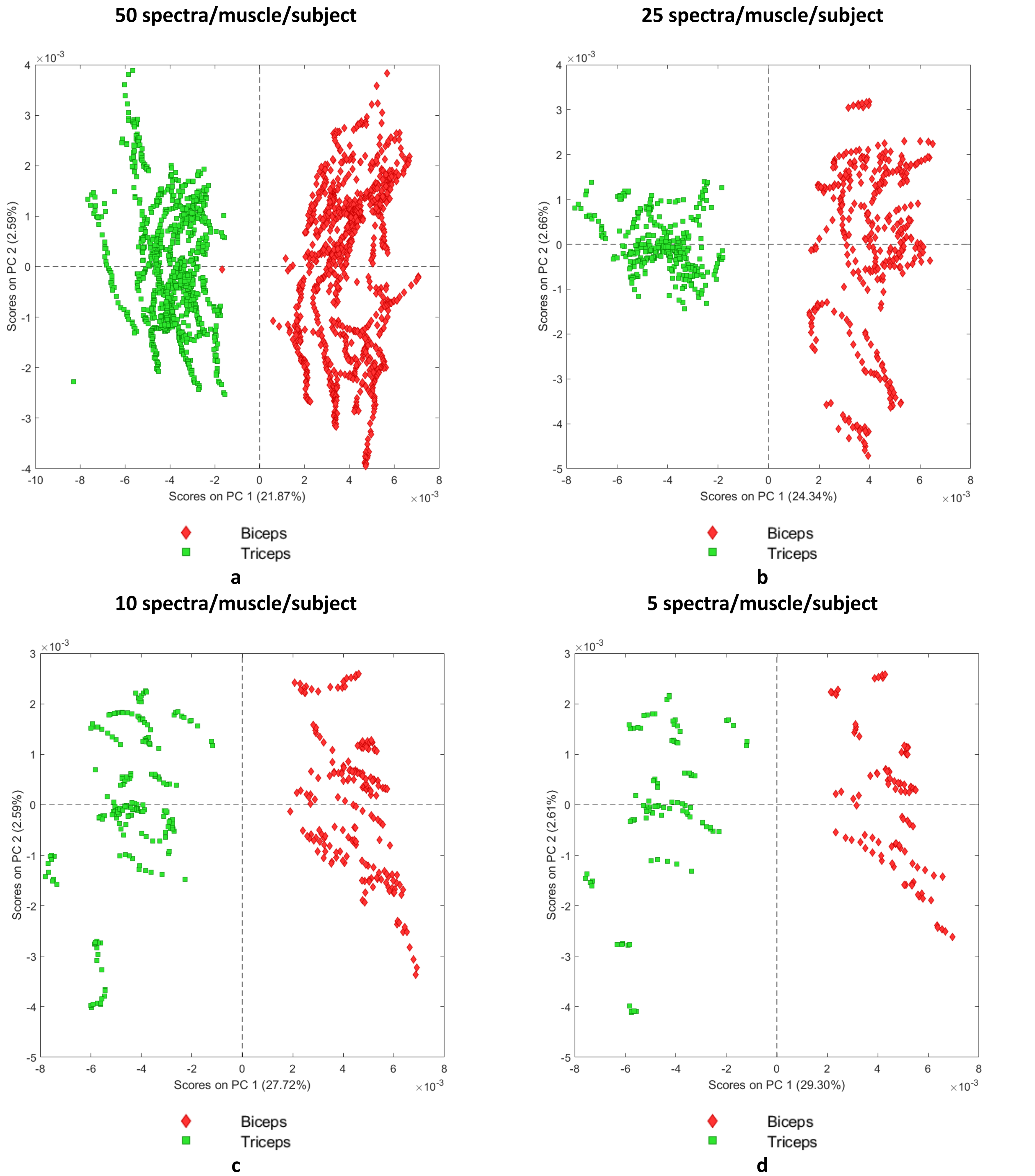

Figure S11. Principal component analysis score plot performed on biceps and triceps reflectance spectra by varying the number of data collected on each muscle/subject: (a) 50 spectra/muscle/subject, (b) 25 spectra/muscle/subject, (c) 10 spectra/muscle/subject and (d) 5 spectra/muscle/subject.

The dataset used for this test is hosted in public repository.
Repository name: Bonifazi, Giuseppe; Currà, Antonio; Gasbarrone, Riccardo; Trompetto, Carlo; Fattapposta, Francesco; Pierelli, Francesco; Missori, Paolo; Serranti, Silvia (2020), “A dataset of Visible – Short Wave InfraRed reflectance spectra collected in-vivo on the dorsal and ventral aspect of arms”, Mendeley Data, v1.
Direct URL to data: http://dx.doi.org/10.17632/24pg3ywxs5.1

Supplementary material 5 reference

- Currà A, Gasbarrone R, Trompetto C, Fattapposta F, Pierelli F, Missori P, Bonifazi G, Serranti, S. (2020). A dataset of visible–short wave infrared reflectance spectra collected in–vivo on the dorsal and ventral aspect of arms. Data in Brief, 33, 106480.

Supplementary material 6: confident intervals

Fig. S12 displays the raw reflectance spectra and the average reflectance spectrum with the standard deviation for each of the considered class (normal biceps, affected biceps, unaffected biceps, normal triceps, affected triceps, and unaffected triceps of the main dataset). Coefficient of variation were evaluated for each group of spectra, as reported in Table S5.

Raw reflectance spectra | **Average reflectance spectrum and SD**

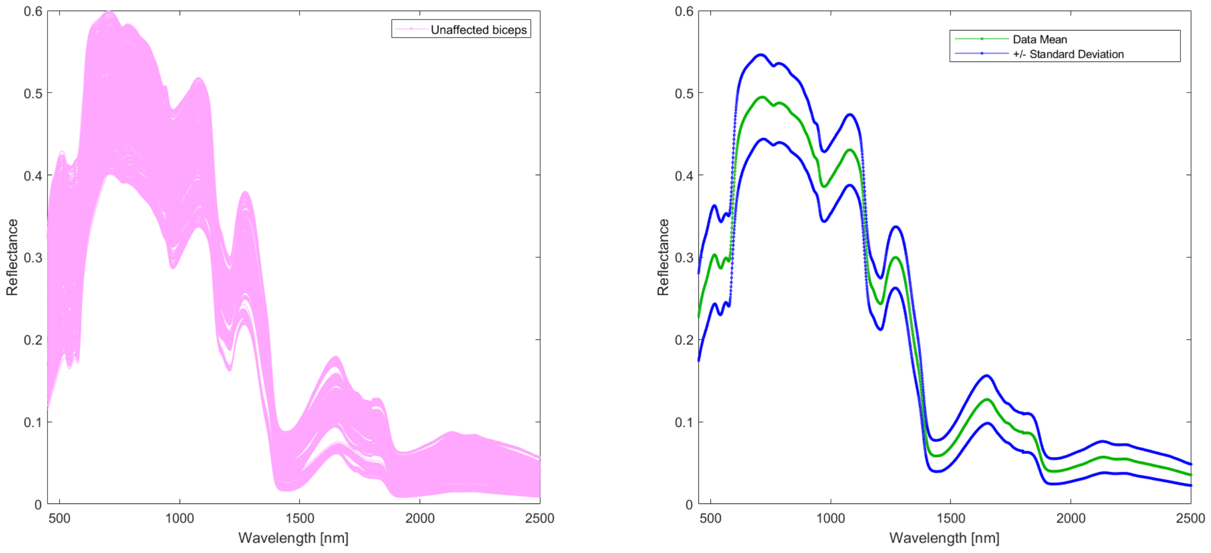

a

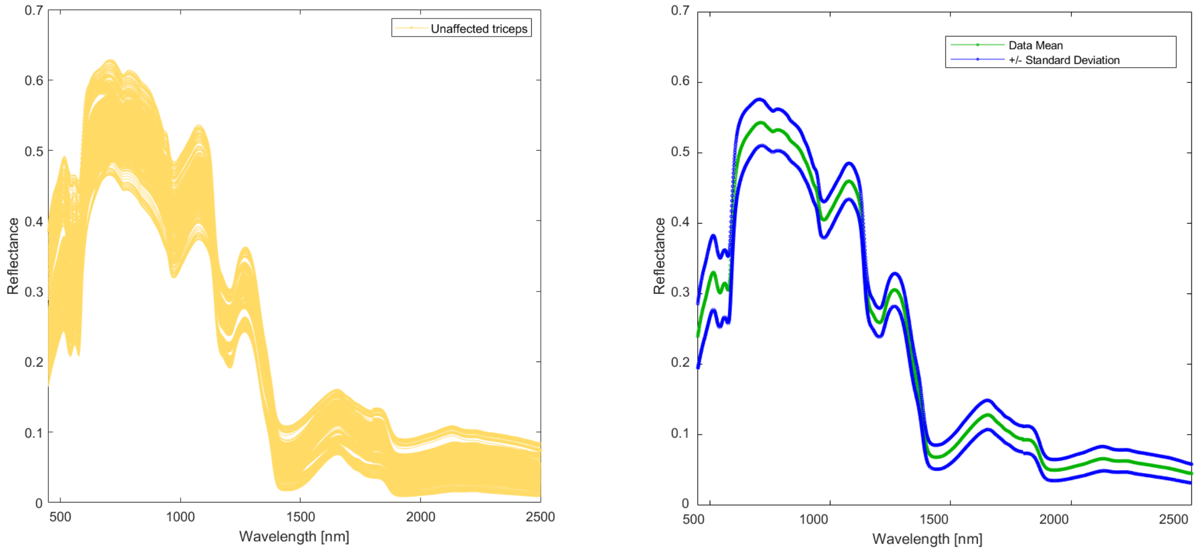

b

Raw reflectance spectra **Average reflectance spectrum + SD**

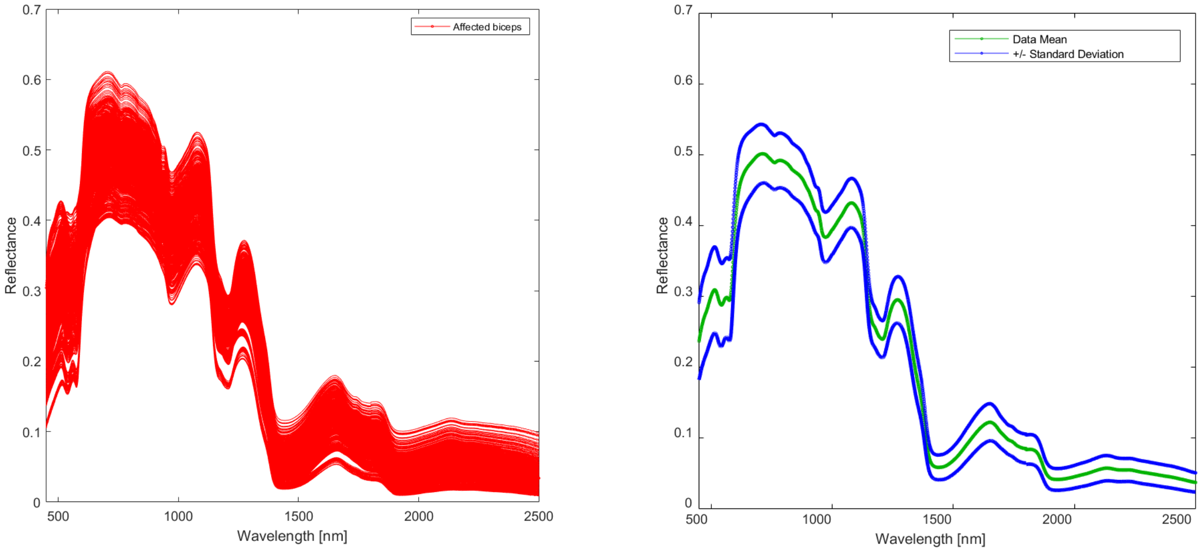

c

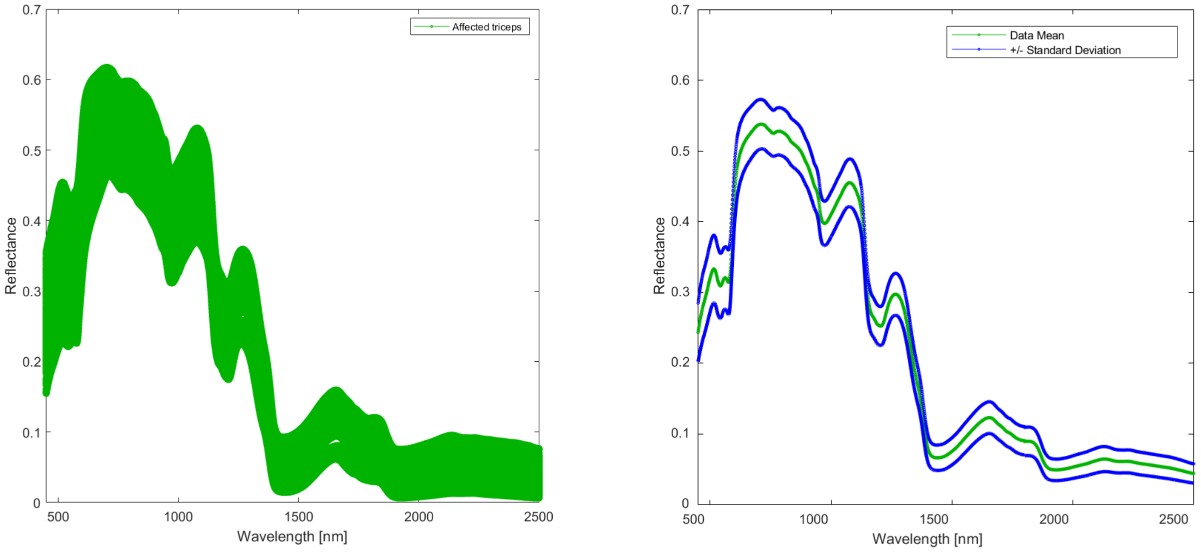

d

Raw reflectance spectra **Average reflectance spectrum + SD**

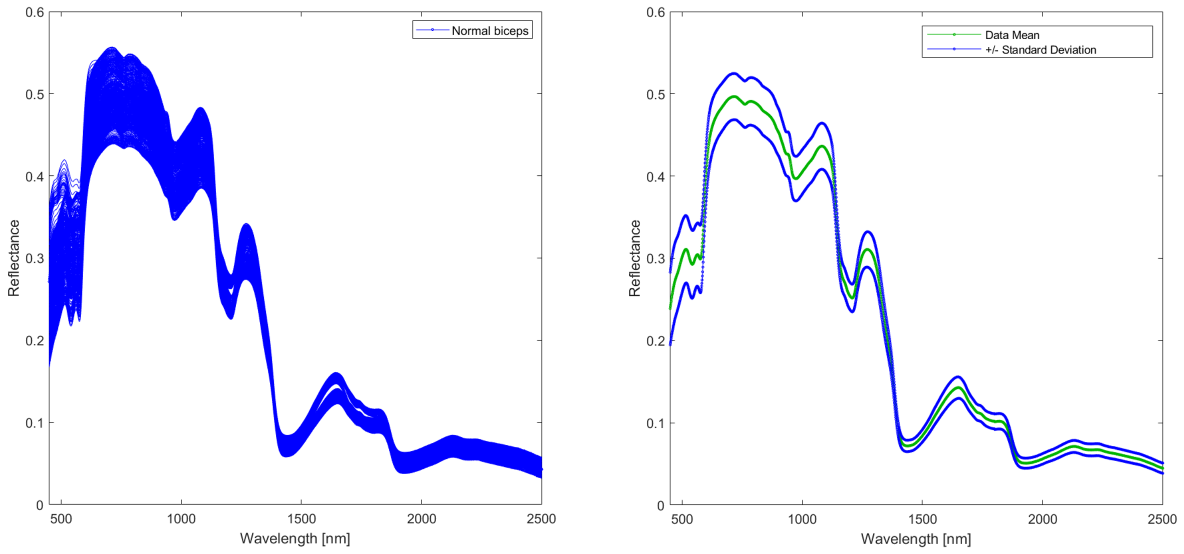

e

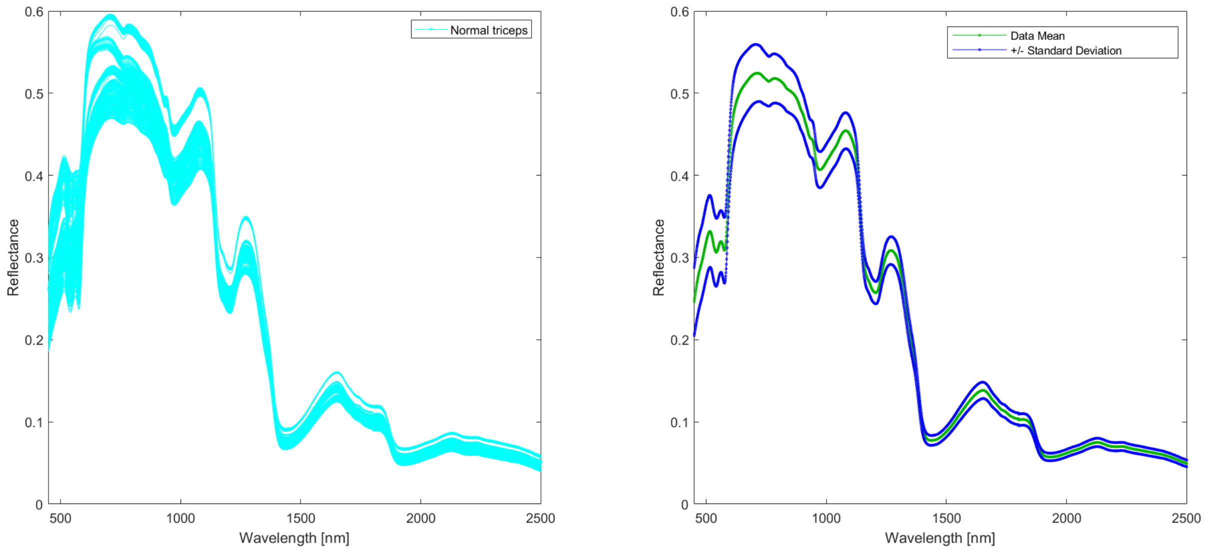

f

Figure S12. Raw reflectance spectra and average reflectance spectra plus the standard deviation for: (a) unaffected biceps, (b) unaffected triceps, (c) affected biceps, (d) affected triceps, (e) normal biceps and (f) normal triceps.

Table S5. Coefficient of variations (CV) of the spectra for the classes: unaffected biceps, unaffected triceps, affected biceps, affected triceps, normal biceps and normal triceps.

	Affected biceps	*Affected triceps*	*Normal biceps*	*Normal triceps*	*Unaffected biceps*	*Unaffected triceps*
Number of spectra	1150	1150	400	400	1150	1150
min CV	0.08	0.06	0.06	0.05	0.10	0.05
max CV	0.37	0.31	0.31	0.24	0.50	0.32
mean CV	0.22	0.19	0.10	0.08	0.23	0.17
